# Lysyl hydroxylase 3–mediated post-translational modifications are required for proper biosynthesis of collagen α1α1α2(IV)

**DOI:** 10.1016/j.jbc.2022.102713

**Published:** 2022-11-17

**Authors:** Yoshihiro Ishikawa, Yuki Taga, Thibault Coste, Sara F. Tufa, Douglas R. Keene, Kazunori Mizuno, Elisabeth Tournier-Lasserve, Douglas B. Gould

**Affiliations:** 1 Department of Ophthalmology, University of California San Francisco, School of Medicine, California, USA; 2 Nippi Research Institute of Biomatrix, Ibaraki, Japan; 3 Université Paris Cité, Inserm Neurodiderot, AP-HP Paris, France; 4 Research Department, Shriners Hospital for Children, Portland, Oregon, USA; 5 Department Anatomy, Cardiovascular Research Institute, Bakar Aging Research Institute, and Institute for Human Genetics, University of California, San Francisco, California, USA

**Keywords:** collagen, post-translational modification, hydroxylase, glycosylation, basement membrane, AAA, amino acid analyses, AHA, l-azidohomoalanine, DMEM, Dulbecco's modified Eagle's medium, EDS, Ehlers–Danlos syndrome, ER, endoplasmic reticulum, gRNA, guide RNA, HSP47, heat shock protein 47, Hyl, hydroxylysine, 4Hyp, 4-hydroxyproline, ICH, intracerebral hemorrhage, LH3, lysyl hydroxylase 3, MEF, mouse embryonic fibroblast, MS, mass spectrometry, OI, osteogenesis imperfecta, P4H, prolyl 4-hydroxylase, PTM, post-translational modification, PVDF, polyvinylidene difluoride, SI-collagen, stable isotope–labeled collagen, SPR, surface plasmon resonance

## Abstract

Collagens are the most abundant proteins in the body and among the most biosynthetically complex. A molecular ensemble of over 20 endoplasmic reticulum resident proteins participates in collagen biosynthesis and contributes to heterogeneous post-translational modifications. Pathogenic variants in genes encoding collagens cause connective tissue disorders, including osteogenesis imperfecta, Ehlers–Danlos syndrome, and Gould syndrome (caused by mutations in *COL4A1* and *COL4A2*), and pathogenic variants in genes encoding proteins required for collagen biosynthesis can cause similar but overlapping clinical phenotypes. Notably, pathogenic variants in lysyl hydroxylase 3 (LH3) cause a multisystem connective tissue disorder that exhibits pathophysiological features of collagen-related disorders. LH3 is a multifunctional collagen-modifying enzyme; however, its precise role(s) and substrate specificity during collagen biosynthesis has not been defined. To address this critical gap in knowledge, we generated LH3 KO cells and performed detailed quantitative and molecular analyses of collagen substrates. We found that LH3 deficiency severely impaired secretion of collagen α1α1α2(IV) but not collagens α1α1α2(I) or α1α1α1(III). Amino acid analysis revealed that LH3 is a selective LH for collagen α1α1α2(IV) but a general glucosyltransferase for collagens α1α1α2(IV), α1α1α2(I), and α1α1α1(III). Importantly, we identified rare variants that are predicted to be pathogenic in the gene encoding LH3 in two of 113 fetuses with intracranial hemorrhage—a cardinal feature of Gould syndrome. Collectively, our findings highlight a critical role of LH3 in α1α1α2(IV) biosynthesis and suggest that LH3 pathogenic variants might contribute to Gould syndrome.

Collagens are the most abundant proteins in the human body and comprise 28 members encoded by 46 genes (1, 2). Pathogenic variants in genes encoding collagens cause a wide range of disorders affecting almost all tissues and organs (3, 4). Type I, type III, and type V collagens form fibrils contributing to structural frameworks and pathogenic variants in these collagens cause connective tissue disorders—osteogenesis imperfecta (OI) and Ehlers–Danlos syndrome (EDS)—that affect fibril-rich tissues, such as bone, skin, and vascular structures (5, 6, 7, 8, 9, 10). In contrast, type IV collagens form flexible networks that are present in the basement membranes of all tissues. Pathogenic variants in type IV collagens cause Alport syndrome (*COL4A3*, *COL4A4*, and *COL4A5*), which primarily affects the glomerular basement membrane (11, 12) and Gould syndrome (*COL4A1* and *COL4A2*), a congenital multisystem disorder that includes highly variable cerebrovascular disease, pediatric epilepsy, ocular anterior segment dysgenesis, skeletal myopathy, and nephropathy (13, 14, 15, 16, 17, 18).

Collagen biosynthesis is a complex and highly orchestrated process that occurs in the endoplasmic reticulum (ER) and is facilitated by a molecular ensemble (19) of over 20 proteins that participate in four major steps: post-translational modifications (PTMs) of individual collagen chains, trimer assembly, triple helix formation, and secretion (19, 20, 21). Although most components of the molecular ensemble are shared among collagens, the collagen-modifying enzymes differ between collagens (22, 23, 24), suggesting subtype specialization of the molecular ensemble (24, 25). Moreover, the molecular ensemble and PTMs for type I collagen, the most extensively characterized collagen, show that the PTM profile is also tissue specific (24, 26). PTMs on the collagen triple helix are classified into three types of primary modifications to prolyl or lysyl residues and two types of secondary modifications to hydroxylysines. Prolyl modifications include 4-hydroxyproline (4Hyp) and 3-hydroxyproline that are generated by prolyl 4 hydroxylases and prolyl 3 hydroxylases, respectively (27). Approximately 40% or more of the prolines are modified to 4Hyp (22, 24, 28), which provides thermal stability to the collagen triple helix (29, 30, 31). In contrast, less than 5% of the prolines are modified to 3-hydroxyproline (28, 32), which is proposed to mediate protein–protein interactions (32, 33). Primary modification of lysine to hydroxylysine (Hyl) is attributed to lysyl hydroxylases (LHs), (27) and secondary modifications of Hyl include two successive and coordinated *O*-glycosylation steps. Hyl is required for subsequent galactose attachment to make galactosyl Hyls, which are further modified by glucose attachment to make glucosyl galactosyl Hyls. This sequence of modifications from lysine, to Hyl, to galactosyl Hyl and glucosyl galactosyl Hyl occurs in the ER during collagen biosynthesis (34, 35).

In the case of OI and EDS, it is well documented that pathogenic variations in genes encoding collagens and genes encoding proteins required for collagen biosynthesis lead to similar clinical outcomes (5, 6, 7, 8, 9, 10). A similar paradigm may also exist for Gould syndrome as pathogenic variants in glycosyltransferase 25 domain containing 1 (GLT25D1), encoded by collagen β (1-O) galactosyltransferase 1 (*COLGALT1*) cause musculoskeletal (36) and cerebrovascular (37, 38) manifestations similar to those observed in individuals with *COL4A1* and *COL4A2* pathogenic variants. Notably, because molecular ensemble proteins can be multifunctional and employed across diverse collagen types, pathogenic variants in encoding genes often cause phenotypes that blend across different clinical categories. An example being LH3, encoded by procollagen-lysine, 2-oxoglutarate 5-dioxygenase 3 (*PLOD3*), whereby pathogenic variants cause connective tissue phenotypes that overlap with Stickler-like syndrome and epidermolysis bullosa (39, 40, 41, 42). Indeed, clinical features of patients with biallelic loss of function *PLOD3* variants include craniofacial dysmorphisms, skeletal and ocular disorders, sensorineural hearing loss, and variable skin manifestations. Interestingly, in addition to clinical manifestations overlapping Stickler-like syndrome and epidermolysis bullosa, cerebral hemorrhages have been reported in three cases with *PLOD3* variants (39, 43). LH3 is proposed to have substrate preference for heavily glycosylated collagens (44, 45, 46) such as type IV collagen (28, 32), raising the possibility that *PLOD3* pathogenic variants could also cause Gould syndrome. Supporting this, *Plod3* deficiency in the mouse lead to basement membrane disruptions caused by intracellular accumulation and impaired secretion of type IV collagen that had lower molecular mass suggesting incomplete PTMs (45). Importantly, according to the peptide-based *in vitro* enzymatic analyses, LH3 is striking in that it is a multifunctional enzyme that can execute all the enzymatic roles required for fully modifying lysine to glucosyl galactosyl Hyl—LH, galactosyltransferase, and glucosyltransferase (39, 44, 47, 48, 49, 50, 51). However, detailed quantitative PTM analysis has never been conducted for any type of collagen in the context of LH3 deficiency. Thus, in this study, we used CRISPR-mediated gene editing to knockout LH3 both in PFHR9 cells and mouse embryonic fibroblasts (MEFs) and performed a multifaceted investigation including cellular biochemistry, structural assessment, and quantitative PTM characterization for collagen α1α1α2(I), α1α1α1(III), and α1α1α2(IV) to elucidate the role of LH3 in the collagen molecular ensemble. Furthermore, since perinatal cerebral hemorrhage is a cardinal feature of Gould syndrome, we performed a genetic screen for *PLOD3* variants in a fetal stroke cohort that was negative for *COL4A1* and *COL4A2* pathogenic variants.

## Results

### LH3 is required for secretion of collagen α1α1α2(IV) but not collagens α1α1α2(I) or α1α1α1(III)

**Figure 1:**
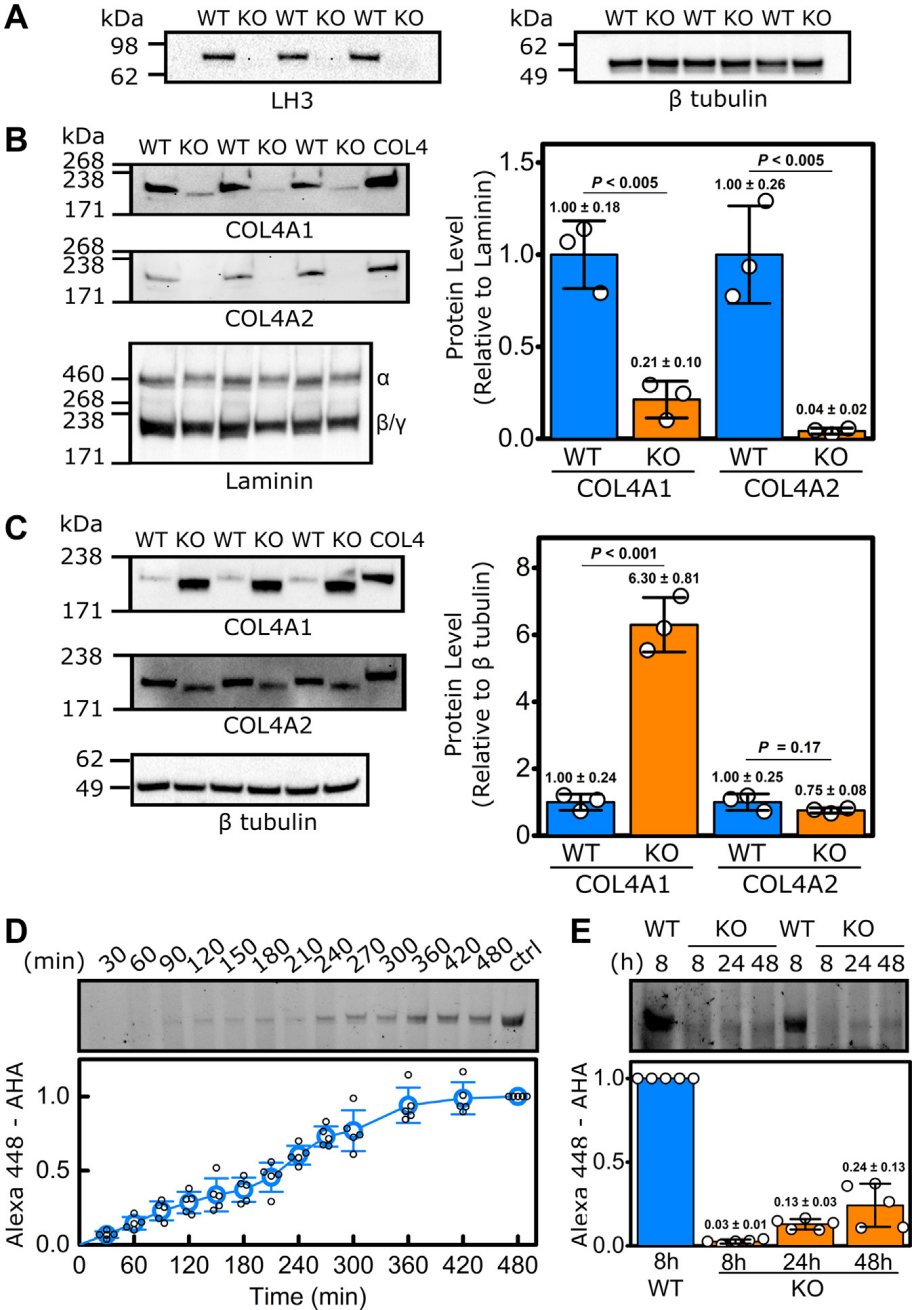
**LH3 deficiency in PFHR9 cells impairs collagen α1α1α2(IV) secretion.***A*, semiquantitative Western blots showing successful ablation of LH3 in PFHR9 cells following CRISPR/Cas9-mediated LH3 inactivation (LH3 KO PFHR9 cells). β-tubulin was used as a loading control. *B*, semiquantitative Western blots and quantification of COL4A1 and COL4A2 levels in the conditioned cell culture medium showing reduced secretion of COL4A1 and COL4A2 in LH3 KO PFHR9 cells (KO) compared with their WT controls. Laminin α/β/γ was used as a loading control. *C*, semiquantitative Western blots and quantification of COL4A1 and COL4A2 levels in cell lysates showing intracellular COL4A1 accumulation in LH3 KO PFHR9 cells. β-tubulin was used as a loading control. COL4 indicates control-purified collagen α1α1α2(IV). The quantification data in *B* and *C* are shown as fold relative to WT levels and presented as means ± SD with individual data points representing independent preparations of culture medium and cell lysate and (n = 3). *D*, representative SDS-PAGE gel (*top*) and quantification (*bottom*) of time-dependent extracellular collagen α1α1α2(IV) levels in WT PFHR9 cells measured by AHA-Alexa Fluor pulse-chase labeling. “ctrl” indicates purified AHA-incorporated collagen α1α1α2(IV). The quantification of AHA-incorporated collagen α1α1α2(IV) was set to 1.0 at 480 min (n = 5). *E*, representative SDS-PAGE gel (*top*) and quantification (*bottom*) of time-dependent extracellular collagen α1α1α2(IV) levels for LH3 KO PFHR9 cells measured by AHA-Alexa Fluor pulse-chase labeling, demonstrating significantly impaired collagen α1α1α2(IV) secretion in LH3 KO PFHR9 cells compared with their WT AHA-incorporated collagen α1α1α2(IV) controls at 8 h (n = 5). Source data files for the figure: The uncropped images of Western blots used in the figure are presented in Fig. S2; generation of the purified AHA-incorporated collagen α1α1α2(IV) used as a control in the figure is presented in Fig. S3; and the value of AHA-incorporated collagen α1α1α2(IV) at each time points is shown in Table S1. AHA, l-azidohomoalanine; LH3, lysyl hydroxylase 3.

To explore the role of LH3 in the molecular ensemble for type IV collagen, we used CRISPR-mediated gene editing to knockout LH3 in PFHR9 cells (Figs. 1*A*, S1 and S2*A*). We selected PFHR9 cells because they are well characterized and secrete large amounts of collagen α1α1α2(IV) because of a duplication of the *Col4a1/Col4a2* locus (28, 32, 52). Western blot analysis revealed a significant reduction in COL4A1 and COL4A2 protein levels in the conditioned culture medium of LH3 KO PFHR9 cells (Figs. 1*B* and S2*B*) and a concomitant increase in intracellular COL4A1 protein levels (Figs. 1*C* and S2*C*), indicating that LH3 deficiency impairs collagen α1α1α2(IV) secretion. To quantitively evaluate the effect of LH3 KO on collagen α1α1α2(IV) biosynthesis, we tested the rate of secretion using pulse-chase labeling with the methionine analog l-azidohomoalanine (AHA) (53, 54). Although this approach is well established for collagen α1α1α2(I), precise measurements of collagen α1α1α2(IV) secretion have not been carefully determined (55, 56, 57, 58). AHA pulse-chase analysis in WT PFHR9 cells showed that newly synthesized collagen α1α1α2(IV) takes approximately 7 to 8 h to be secreted (Fig. 1*D*, Table S1, and Fig. S3) compared with approximately 2 h for collagen α1α1α2(I) (59, 60). However, in LH3 KO PFHR9 cells, collagen α1α1α2(IV) secretion was significantly delayed, and only one-fourth of newly synthesized collagen α1α1α2(IV) was secreted even after 48 h in culture (Fig. 1*E* and Table S1).

**Figure 2:**
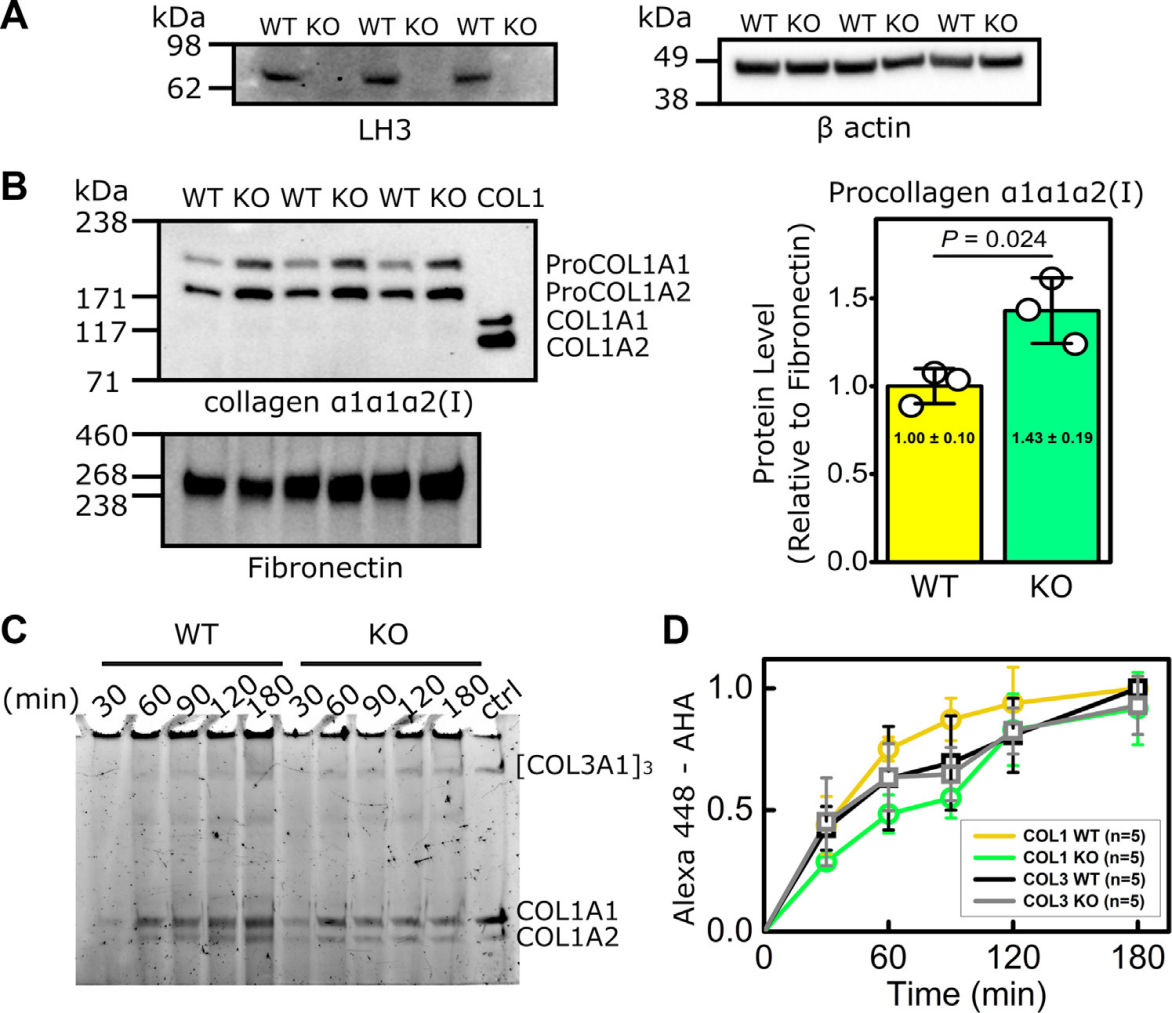
**LH3 deficiency in MEFs does not impair biosynthesis of collagen α1α1α2(I) or collagen α1α1α1(III).***A*, semiquantitative Western blots showing successful ablation of LH3 in MEFs following CRISPR/Cas9-mediated LH3 inactivation (LH3 KO MEFs). β-actin was used as a loading control. *B*, semiquantitative Western blots and quantification of collagen α1α1α2(I) in conditioned cell culture medium showing increased secretion of ProCOL1A1 and ProCOL1A2 in LH3 KO MEFs (KO) compared with their WT controls. Fibronectin was used as a loading control. COL1 indicates purified pepsin-treated collagen α1α1α2(I). The quantification data in *B* are shown as fold relative to WT levels and presented as means ± SD with individual data points representing independent preparations of culture medium (n = 3). *C*, representative SDS-PAGE gel. *D*, quantification of time-dependent extracellular levels of collagens α1α1α2(I) and α1α1α1(III) measured by AHA-Alexa Fluor pulse-chase labeling, demonstrating comparable secretion rate between LH3 KO and WT MEFs. “ctrl” indicates purified AHA-incorporated collagens α1α1α2 (I) and α1α1α1(III). WT AHA incorporation signal was set to 1.0 at 180 min (n = 5). Source data files for the figure: The uncropped images of Western blots used in the figure are presented in Fig. S5; generation of the purified AHA-incorporated pepsin-treated collagens α1α1α2(I) and α1α1α1(III) mixture used as a control in *C* is presented in Fig. S6; the value of AHA-incorporated collagens α1α1α2(I) and α1α1α1(III) at each time points is shown in Table S2. AHA, l-azidohomoalanine; LH3, lysyl hydroxylase 3; MEF, mouse embryonic fibroblast.

Because PFHR9 cells produce very low levels of fibrillar collagens, we also used CRISPR-mediated gene editing to knockout LH3 in MEFs to test the effect of LH3 deficiency on collagen α1α1α2(I) and collagen α1α1α1(III) biosynthesis (Figs. 2*A*, S4 and S5*A*). Notably, Western blot analysis showed that the levels of extracellular collagen α1α1α2(I) were increased in LH3 KO MEFs compared with their WT controls (Figs. 2*B* and S5*B*). Since the signals of α2(I) chain were stronger than that of α1(I) chain in Western blot (Figs. 2*B* and S5*B*), we quantitively tested the effect of LH3 KO on collagen secretion rates in MEFs. AHA pulse-chase labeling showed that the secretion rates of fibrillar collagens α1α1α2(I) and α1α1α1(III) were comparable between WT and LH3 KO MEFs, although delays were observed at early time points for collagen α1α1α2(I) (Fig. 2, *C* and *D*, Table S2 and Fig. S6). Collectively, these data suggest that LH3 is critical for biosynthesis of collagen α1α1α2(IV) but not collagens α1α1α2(I) and α1α1α1(III).

### LH3 is required for collagen α1α1α2(IV) to form triple helices and higher order oligomeric structures

**Figure 3:**
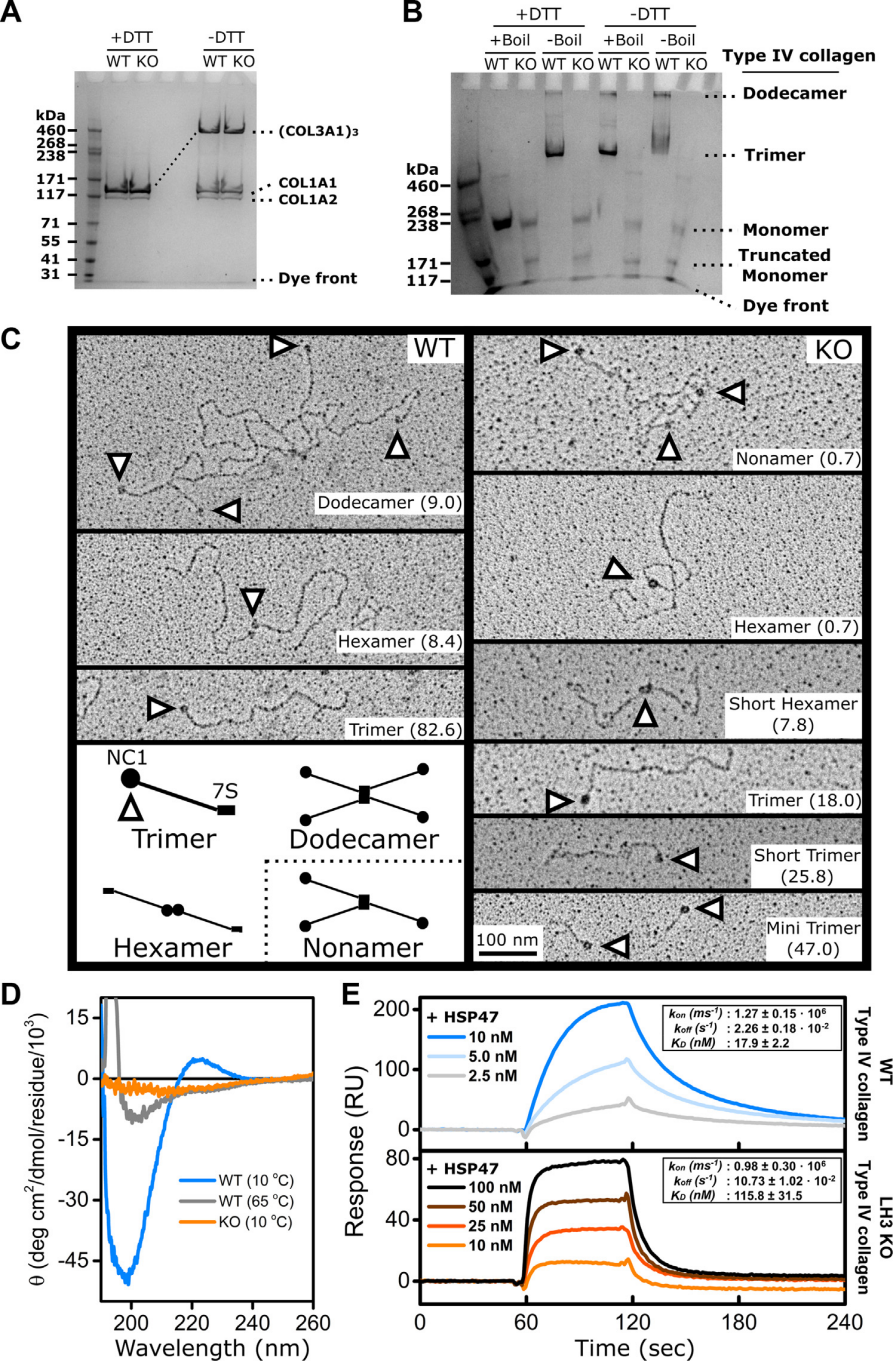
**LH3 deficiency impairs collagen α1α1α2(IV) oligomerization and triple helix formation.***A*, representative Coomassie blue–stained 3 to 8% Tris–acetate gel showing pepsin-treated collagens α1α1α2(I) and α1α1α1(III) purified from conditioned culture medium of WT and LH3 KO MEFs. *B*, representative Coomassie blue–stained 3.5% SDS-agarose gel showing collagen α1α1α2(IV) purified from conditioned culture medium of WT and LH3 KO PFHR9 cells. *C*, representative transmission electron rotary shadow microscopy images showing structural alterations of collagen α1α1α2(IV) purified from conditioned culture medium of WT and LH3 KO PFHR9 cells. *Arrowheads* point to globular NC1 domains. Diagram at the *bottom left* illustrates normal collagen α1α1α2(IV) network formation. The frequency of each molecular species is indicated in parentheses (%). *D*, CD spectra of purified collagen α1α1α2(IV) from conditioned culture medium of WT and LH3 KO PFHR9 cells under native (10 ^°^C) and denaturing (65 ^°^C) conditions. *E*, surface plasmon resonance analysis showing concentration-dependent binding kinetics between HSP47 and collagen α1α1α2(IV) purified from conditioned culture medium of WT and LH3 KO PFHR9 cells. Each *curve* represents the average of a minimum of three measurements. The binding constants and affinities are shown as mean ± SD in the *inset*. LH3, lysyl hydroxylase 3; MEF, mouse embryonic fibroblast.

To molecularly characterize the effects of LH3 deficiency on collagens, we purified collagens α1α1α2(I), α1α1α1(III), and collagen α1α1α2(IV) from the conditioned culture medium of MEFs and PFHR9 cells, respectively (Fig. S7, *A* and *B*). Consistent with the Western blot and AHA pulse-chase data, there was no difference between collagens α1α1α2(I) and α1α1α1(III) produced by WT and LH3 KO MEFs (Fig. 3*A*). In contrast, collagen α1α1α2(IV) from LH3 KO PFHR9 cells did not show the higher order collagen α1α1α2(IV) structures that were observed in collagen α1α1α2(IV) from WT PFHR9 cells suggesting an absence of collagen α1α1α2(IV) disulfide crosslinking (Fig. S7*B*). We next used SDS-agarose gel electrophoresis to resolve the oligomeric state of collagen α1α1α2(IV). Consistent with previous reports (52, 56), the protein banding pattern observed in WT PFHR9 cells showed that most collagen α1α1α2(IV) was present as trimers or dodecamers unless denatured under reducing conditions when they were resolved as monomers (Fig. 3*B*). In contrast, most of the collagen α1α1α2(IV) isolated from LH3 KO PFHR9 cells was monomeric and failed to form higher order structures (Fig. 3*B*). Interestingly, we detected a lower molecular weight band in the samples isolated from LH3 KO PFHR9 cells on both SDS-agarose and SDS-PAGE gels (Figs. 3*B* and S7*C*), which was identified as truncated COL4A1 and COL4A2 proteins by LC–MS analysis (Fig. S7*D*). To better characterize collagen α1α1α2(IV) oligomeric structures and network formation, we performed rotary shadow electron microscopy on samples isolated from WT and LH3 KO PFHR9 cells. During type IV collagen network formation, two collagen α1α1α2(IV) heterotrimers associate with each other by way of head-to-head interactions of their carboxy terminal NC1 domains (hexamers) (61) and four collagen α1α1α2(IV) heterotrimers interact by way of lateral and antiparallel interactions of their amino-terminal 7S domains (dodecamers) (62). Electron micrographs from WT PFHR9 cells showed that over 80% of the collagen α1α1α2(IV) was trimeric, but we also found frequent examples of hexamers (∼8.5%) and dodecamers (∼9.0%; Fig. 3*C*). In contrast, we found reduced proportions of normal trimers and hexamers and did not observe dodecamers in samples from LH3 KO cells. Instead, we detected shortened versions of collagen α1α1α2(IV) hexamers (7.8%) and trimers (72.8%) that appeared truncated at the amino termini and corroborate the SDS-gel electrophoreses and LC–MS data (Figs. 3, *B*, *C*, and S7, *B*–*D*).

To evaluate the secondary structure of the collagen α1α1α2(IV) triple helix, we measured CD spectra of collagen purified from WT or LH3 KO PFHR9 cells (Fig. 3*D*). At 10 °C, collagen α1α1α2(IV) isolated from WT cells showed the characteristic triple helical spectra with negative and positive peaks at 200 and 220 nm, respectively (63), and these peaks were lost following heat denaturation at 65 °C. In contrast, even at 10 °C, collagen α1α1α2(IV) isolated from LH3 KO PFHR9 cells resembled denatured WT collagen α1α1α2(IV) indicating that collagen α1α1α2(IV) produced by LH3 KO cells did not form stable triple helical helices. The triple helical structure is required for binding of the collagen molecular chaperone heat shock protein 47 (HSP47) (64), which plays a critical role in collagen trafficking by anchoring collagens to TANGO1 at ER exit sites to continue along the secretory pathway to the Golgi (65). To test whether HSP47 could bind purified collagen α1α1α2(IV), we performed surface plasmon resonance (SPR) analysis. HSP47 interacted with immobilized collagen α1α1α2(IV) from WT PFRH9 cells in a concentration-dependent manner (Fig. 3*E*). Although HSP47 also interacted with collagen α1α1α2(IV) from LH3 KO PFRH9 cells, the binding kinetics were altered with lower binding affinity and increased dissociation constant.

### LH3 is a selective LH but a general collagen glucosyltransferase

**Figure 4:**
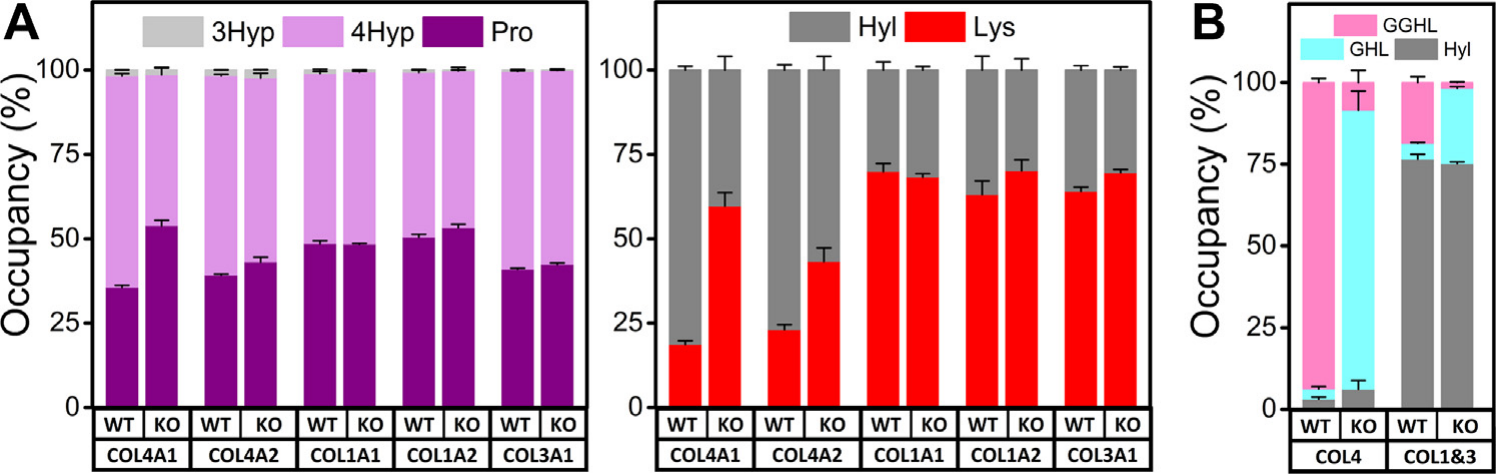
**LH3 is a selective lysyl hydroxylase and general collagen glycosyltransferase.***A*, acid hydrolysis amino acid analysis. Histograms showing the occupancy of post-translational modifications in proline (3Hyp + 4Hyp + Pro = 100%) and lysine (Hyl + Lys = 100%) in individual alpha chains of collagens α1α1α2(IV), α1α1α2(I), and α1α1α1(III). *B*, alkaline hydrolysis amino acid analysis. Histogram showing the occupancy of *O*-glycosylation attached to Hyl (GGHL + GHL + Hyl = 100) in purified collagens α1α1α2(IV) and mixture of collagens α1α1α2(I) and α1α1α1(III). Source data files for the figure: The value of each amino acid, the number of biological replicates, and detailed statistical analyses for data shown in the figure are presented in Table S3 and Fig. S8, respectively. GGHL, glucosyl galactosyl Hyl; GHL, galactosyl Hyl; Hyl, hydroxylysine; 3Hyp, 3-hydroxyproline; 4Hyp, 4-hydroxyproline; LH3, lysyl hydroxylase 3; Lys, lysine; Pro, proline.

PTMs are important for the structural integrity of collagen triple helices. To understand how LH3 deficiency impacted collagen PTMs, we quantified prolyl hydroxylation, lysyl hydroxylation, and *O*-glycosylated Hyl using amino acid analyses (AAA) combined with LC–MS. First, we performed acid hydrolysis AAA (which removes sugar groups) of individual collagen α chains from WT or LH3 KO cells. We found that prolyl 3 hydroxylation, which occurs infrequently, was largely unchanged by LH3 deficiency across COL1A1, COL1A2, COL3A1, COL4A1, and COL4A2 proteins and that prolyl 4 hydroxylation was reduced for COL4A1 but not for the other α chains (Fig. 4*A* and Table S3). In contrast, we found more unmodified lysines (significantly fewer hydroxylysines), on COL4A1 and COL4A2 proteins isolated from LH3 KO PFHR9 cells compared with WT PFHR9 cells. The levels of lysine/Hyl for COL1A1, COL1A2, and COL3A1 isolated from MEFs were largely unchanged, suggesting that the LH function of LH3 is selective for collagen α1α1α2(IV) (Fig. 4*A* and Table S3). Next, we used alkaline hydrolysis AAA (which preserves sugar attachments) combined with LC–MS to compare the extent of galactosyl Hyl and glucosyl galactosyl Hyl on collagens between WT and LH3 KO cells. Type IV collagen is highly glycosylated, and Hyl residues from collagen α1α1α2(IV) produced by WT PFHR9 cells were almost fully modified to glucosyl galactosyl Hyl (Figs. 4*B* and S8). In contrast, collagen α1α1α2(IV) derived from LH3 KO PFHR9 cells had high occupancy of galactosyl Hyl but not glucosyl galactosyl Hyl suggesting that in addition to its role as an LH, LH3 also participates in glucosyl, but not galactosyl, modifications. As reported previously (44, 66), glucosyl galactosyl Hyl was also reduced in collagen α1α1α2(I) and collagen α1α1α1(III) suggesting that LH3 is also involved in glucosyl modifications of fibrillar collagens (Figs. 4*B* and S8). Taken together, these data suggest that LH3 has selective LH activity but general collagen glucosyltransferase activity.

### Rare *PLOD3* variants in fetuses with intracerebral hemorrhage

**Figure 5:**
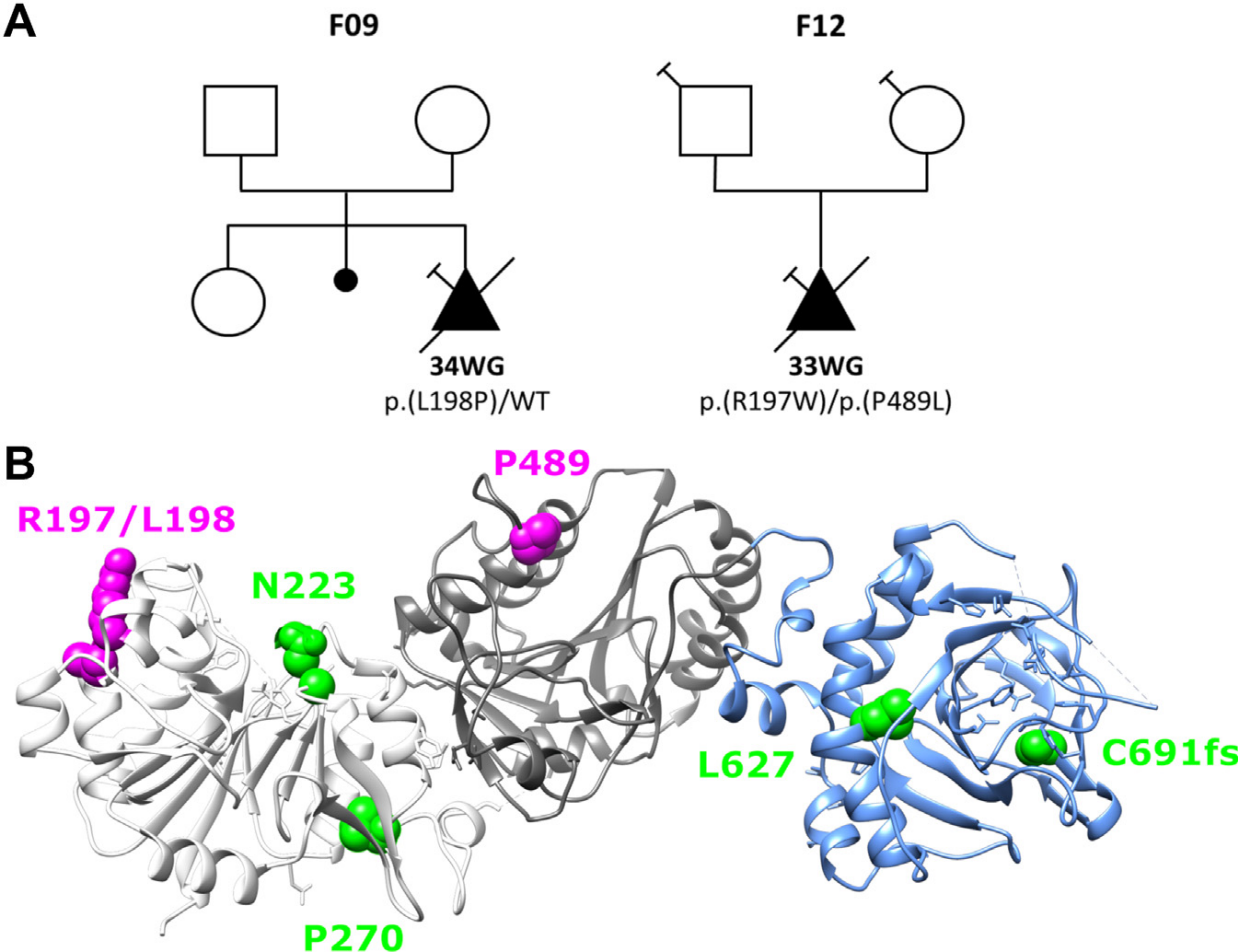
**Identification of four rare *PLOD3* variants in fetal ICH cases.***A*, genealogical trees of the three families with *PLOD3* variants. *Square* = male, *circle* = female, *triangle* = pregnancy not carried to term, *black filled symbol* = affected individual, *empty symbol* = clinically healthy relative, *diagonal black line* = deceased fetuses, and *syringe symbol* = blood sampled individual. The age of termination of pregnancy is indicated under each case. *B*, the crystal structure of the LH3 shows three different domains: catalytic glycosyltransferase domain (*white*), accessory glycosyltransferase domain (*gray*), and lysine dioxygenase domain (*light blue*). Missense variants identified in humans are indicated in different colors on the LH3 crystal structure. *Green* indicates previously reported variants have connective disorders resembling Stickler syndrome-like and epidermolysis bullosa. *Magenta* indicates variants identified in this study. ICH, intracerebral hemorrhage; LH3, lysyl hydroxylase 3.

The role for LH3 in collagen α1α1α2(I) and collagen α1α1α1(III) glycosylation is consistent with the identification of *PLOD3* pathogenic variants in individuals with connective tissue disorders (39, 41). However, our data demonstrate that LH3 plays an even greater role in collagen α1α1α2(IV) biosynthesis. Consistent with this finding, *Plod3* deficiency in the mouse caused basement membrane defects (44, 45), suggesting that *PLOD3* pathogenic variants may also contribute to intracerebral hemorrhages (ICHs), which constitute a central pathophysiological hallmark of Gould syndrome caused by *COL4A1* and *COL4A2* pathogenic variants (13, 14, 15, 67, 68, 69, 70, 71). In addition, cerebrovascular hemorrhages have been reported in three patients with a connective tissue disorder caused by biallelic pathogenic variants of *PLOD3* (39, 43). To investigate the potential involvement of *PLOD3* in ICH, we specifically evaluated the presence of *PLOD3* variants in exome sequencing data from a fetal ICH cohort consisting of 113 probands who did not have *COL4A1* or *COL4A2* pathogenic variants. We did not find an enrichment of rare and predicted damaging *PLOD3* qualifying variants between this cohort and the gnomAD control database (Table 1). However, in two unrelated fetuses, we identified three rare (<1/1000) *PLOD3* missense variants—all three were predicted to be damaging by PolyPhen-2 (Fig. 5*A* and Table 2). Fetus F09 was heterozygous for p.L198P variant, and fetus F12 was compound heterozygous for p.R197W and p.P489L. Variants p.R197W and p.L198P are located in the catalytic glycosyltransferase domain close to a poly Asp sequence and predicted to affect the overall structure of active site in the transferase domain (50) (Fig. 5*B*). The p.P489L variant is located in the accessory glycosyltransferase domain and predicted to affect the substrate recognition (Fig. 5*B*). The two fetuses with predicted pathogenic variants had ICH identified on ultrasound follow up during the third trimester of pregnancy. Upon cerebral imaging, they presented a bilateral ventriculomegaly and intraparenchymal lesions (Table 3). Because of the severity of these lesions, the parents opted for termination of pregnancy. The lesions were confirmed by postmortem examination, and the fetuses showed some phenotypic differences. The fetus F12 had intraparenchymal hemorrhagic and ischemic lesions, whereas the fetus F09 had a microcephaly, a very thin corpus callosum, and a white matter atrophy. Neither had bone abnormalities or signs of external visceral malformation.

**Table 1:** Enrichment analysis of rare predicted damaging *PLOD3* variants in fetus cohort *versus* gnomAD cohorts

gnomAD	Number of loss-of-function and missense variants with an MAF ≤1/1000 in *PLOD3* gene	*p*
v2.1 (N = 141,456 individuals)	2160	0.69
v3.1 (N = 76,156 individuals)	1225	0.7

MAF, minor allele frequency.

**Table 2:** Rare variants identified in *PLOD3* gene in the cohort of 113 ICH fetuses

Fetus	HGVSc position (NM_001084.5)	Protein change	Variant class	Status	gnomAD, v.2.1	PolyPhen-2 prediction	ACMG class
					AF (%)		
F09	c.593T>C	p.(L198P)	Missense	HTZ	0.0004	Probably damaging (0.994)	3
F12^a^	c.589C>T	p.(R197W)	Missense	HTZ	0.0012	Possibly damaging (0.735)	3
F12^a^	c.1466C>T	p.(P489L)	Missense	HTZ	0.051	Possibly damaging (0.769)	3

Abbreviations: ACMG, American College of Medical Genetics and Genomics; AF, allele frequency; HTZ, heterozygous.

ClinVar accession numbers: SCV002558784 (p.L198P), SCV002558785 (p.R197W), and SCV002558786 (p.P489L).

a Biallelic variants (inherited from each parent).

**Table 3:** Main clinical and pathological features of ICH-affected fetuses with *PLOD3* variants

Fetus	F09	F12
Sex	Female	Female
Gestation at diagnosis (weeks)	31	30
Brian imaging findings	Microcephaly, ventriculomegaly, interhemispheric cyst, porencephalic cavities, thin corpus callosum	Asymmetric bilateral ventriculomegaly, ischemic–hemorrhagic lesions in the right hemisphere
Outcome	TOP	TOP
Fetal brain examination	Important ventriculomegaly, white matter atrophy, severe microcephaly with diffuse bilateral lesions, absent or very thin corpus callosum	Asymmetric bilateral ventriculomegaly, intraparenchymal hemorrhages with cortical and subcortical ischemic foci
Skeletal X-ray examination	No sign of bone abnormality except for the absence of the 12th right rib	No sign of bone abnormality
Other autopsy anomalies	No sign of external or visceral malformation	No sign of external or visceral malformation

Abbreviation: TOP, termination of pregnancy.

## Discussion

To clarify the role(s) for LH3 in collagen biosynthesis, we generated LH3 KO cells using CRIPSR-mediated gene editing to conduct detailed quantitative and molecular analyses of collagens α1α1α2(I), α1α1α1(III), and α1α1α2(IV) implicated in OI, EDS, and Gould syndrome, respectively. We discovered that LH3 deficiency severely impaired biosynthesis of collagen α1α1α2(IV) but not collagen α1α1α2(I) or collagen α1α1α1(III). Our findings revealed altered PTMs in collagen α1α1α2(IV) produced by LH3 KO PFHR9 cells characterized by reduced and impaired modification of lysine to Hyl and galactosyl Hyl to glucosyl galactosyl Hyl. These PTM alterations prevented proper heterotrimer formation and HSP47 binding, which impaired collagen α1α1α2(IV) secretion. Notably, collagen α1α1α2(IV) that was secreted from LH3 KO PFHR9 cells showed impaired collagen α1α1α2(IV) network formation and failed to make higher order oligomers. In contrast, LH3 deficiency did not impact primary modifications to prolyl and lysyl residues for collagens α1α1α2(I) and α1α1α1(III) but specifically reduced modification of galactosyl Hyl to glucosyl galactosyl Hyl. Thus, we suggest that LH3 may be a selective LH at least for collagen α1α1α2(IV) and that network-forming and fibrillar collagens are both important substrates for LH3 glucosyltransferase activity.

Our results indicate that LH3 plays a critical role for collagen α1α1α2(IV) biosynthesis in PFHR9 cells but not for collagens α1α1α2(I) and α1α1α1(III) in MEFs. Surprisingly, Western blot analysis for PFHR9 cells showed that protein levels of intracellular COL4A2 in KO cells were similar to WT control cells; however, the protein migrated slightly faster and its secretion was significantly impaired (Fig. 1, *B* and *C*), indicating there could be a different quality control mechanism between COL4A1 and COL4A2 proteins. Although the majority of synthesized collagen α1α1α2(IV) was accumulated intracellularly in LH3 KO PFHR9 cells, some collagen α1α1α2(IV) was successfully secreted but did not have stable triple helical structures. Based on the current understanding of fibrillar collagen biosynthesis, this finding is intriguing for two reasons. First, the amount of 4Hyp is essential to the formation of triple helix rigidity (29). Type II collagen containing approximately 30% 4Hyp from *P4ha1*^*+/−*^, *P4ha2*^*−/−*^ mice (*P4ha1* and *P4ha2* encoded prolyl 4-hydroxylase 1 and 2, respectively) decreased its thermal stability compared with a WT control; however, this type II collagen remained capable of forming a triple helical structure (72). Notably, the COL4A1 produced by LH3 KO PFHR cells showed a reduction of 4Hyp (Fig. 4*A*) but still contained around total 45% 4Hyp in the collagen α1α1α2(IV) heterotrimer isolated from LH3 KO PFHR9 cells, which is calculated by the amount of 4Hyp in COL4A1 and COL4A2 (Table S3). Second, the amounts of Hyl do not affect triple helix formation. Collagen α1α1α2(I) containing only 1.5% Hyl can form fully rigid triple helices and secrete into the extracellular matrix properly (24). Also, increased hydroxylysines in collagen α1α1α2(I) do not improve collagen thermal stability (73). Based on these observations, it is tempting to speculate that lysyl hydroxylation and/or the subsequent *O*-glycosylation critically contributes triple helix stability in collagen α1α1α2(IV).

Interestingly, the AHA pulse-chase experiments showed that the secretion rate of collagen α1α1α2(IV) was remarkably slower than that of collagens α1α1α2(I) and α1α1α1(III) (Figs. 1*D* and 2*D*). A significant difference between collagen α1α1α2(IV) and fibrillar collagens (type I, II, III, and V) is the presence of multiple interruptions of the collagenous repeats, which occur more frequently nearer the amino terminus (74). Repeat interruptions, which would be pathogenic if they occurred in fibrillar collagens, may interfere with propagation of triple helix formation, requiring renucleation of the three alpha chains, and likely contribute to the much longer secretion time for collagen α1α1α2(IV) compared with collagens α1α1α2(I) and α1α1α1(III). We hypothesize that the complement of PTMs near repeat interruptions may provide thermal or structural stability required for renucleation and propagation of the collagen α1α1α2(IV) triple helix. Supporting this notion, collagen α1α1α2(IV) from LH3 KO cells appeared shorted on rotary shadow images, which may suggest that the amino termini are disordered and cannot be resolved by electron microscopy. Alternatively, they may be more susceptible to cleavage, leading to a truncated protein of lower molecular weight as demonstrated by SDS gel electrophoreses and LC–MS analysis (Figs. 3, *B*, *C* and S7, *B*–*D*). Structural alterations of the 7S domain at the amino termini could be responsible for the inability to form higher order dodecamer structures in collagen α1α1α2(IV) isolated from LH3 KO PFHR9 cells (Figs. 3, *B*, *C* and S7, *B*–*D*). While the CD spectra showed that the overall secondary structure of collagen α1α1α2(IV) produced by LH3 KO was not fully triple helical (Fig. 3*D*), HSP47 was still able to bind with this collagen α1α1α2(IV). We speculate that this binding occurs on the triple helical carboxyl terminus region and weakly associate with the immature amino-terminal gelatin-like structure (Fig. 3*E*). Interestingly, small amounts of collagen α1α1α2(IV) were secreted from LH3 KO cells without a robust triple helical structure, which raises the question: what are the essential chemical or structural factors for collagen α1α1α2(IV) triple helix formation and for passing through quality control checkpoints in the ER and secretory pathway? Further experiments are required to provide additional mechanistic insights into the role of PTMs in collagen α1α1α2(IV) biosynthesis, secretion, and function(s).

Since our findings establish a critical role for LH3 in collagen α1α1α2(IV) biosynthesis, we predicted that *PLOD3* might lead to clinical manifestations similar to those observed in individuals with *COL4A1* and *COL4A2* pathogenic variants causing Gould syndrome, a multisystem disorder frequently manifesting as fetal and perinatal ICH (13, 14, 15, 67, 68, 69, 70). Previously identified pathogenic *PLOD3* missense variants cause phenotypically overlapping connective disorders including Stickler-like syndrome and epidermolysis bullosa (39, 40, 41, 42). In addition to the clinical features encountered in these two conditions, spontaneous ICH has been reported by Salo *et al.* (39) in two siblings. One of them was a female child with a connective tissue disorder, consisting of craniofacial dysmorphism, sensorineural deafness, and skeletal features, who presented a spontaneous large brain hematoma. Her male sibling was stillborn at 28 weeks of gestation; in addition to skeletal anomalies, autopsy of this fetus showed porencephaly. DNA analysis showed biallelic *PLOD3* variants (p.N223S/p.C691AfsX9) located in a conserved region of the LH3 amino acid sequence responsible for collagen glycosyltransferase and LH activities. Recently, a homozygous *PLOD3* small inframe deletion was shown to cause a cerebral small vessel disease characterized by multiple white matter hypersignals and bleeding foci in an infant with a few dysmorphic features (43). To evaluate the contribution of *PLOD3* pathogenic variants to cerebrovascular manifestations, we analyzed a series of 113 cases of fetal ICH that were negative for *COL4A1* and *COL4A2* pathogenic variants. We identified a rare and predicted damaging heterozygous variant in one fetus (F09) and two distinct biallelic candidate variants in another fetus (F12) (Fig. 5 and Table 2). When comparing the whole cohort of fetuses to control databases, we did not detect an enrichment in rare and predicted damaging *PLOD3* variants; however, this lack of enrichment could be attributable to the high genetic heterogeneity of fetal ICH (71). Additional work is required to establish the causality of the heterozygous *PLOD3* variant present in fetus F09. Indeed, connective tissue disorders associated with *PLOD3* are typically autosomal recessive, and heterozygous parents of affected cases are reported to be asymptomatic. We cannot exclude however that some heterozygous *PLOD3* variants could have dominant negative effects or that they could act as risk factors acting in concert with other triggering events to cause fetal ICH. For fetus F12, who is a compound heterozygote with both variants being predicted to be possibly pathogenic, no tissue was available to evaluate the consequences of the two variants at the protein level. Autopsy data including skeletal X-ray data were normal. Additional *in vitro* experiments are required to determine the causality of these variants, and larger cohorts of fetuses and children with ICH should be analyzed to identify additional cases.

In conclusion, our results suggest that LH3 plays a critical and multifunctional role in collagen α1α1α2(IV) biosynthesis, and that *PLOD3* variants could lead to clinical manifestations associated with *COL4A1* and *COL4A2* pathogenic variants.

## Experimental procedures

### Cell culture

PFHR9 cells (CRL-2423) and MEFs (CRL-1503) were purchased from American Type Culture Collection. These cells were cultured in Dulbecco's modified Eagle's medium (DMEM)/high glucose/pyruvate (Gibco; catalog no.: 11995065) supplemented with 10% (v/v) fetal bovine serum (Atlanta Biologicals), pencillin streptomycin glutamine 100× (Gibco; catalog no.: 10378016), and 5 mM Hepes in the presence of ascorbic acid phosphate (100 μg/ml; Wako Chemicals). After this section, standard DMEM indicates the aforementioned DMEM.

### CRISPR-mediated KO of LH3

LH3 was knocked out in PFHR9 cells and MEFs using two CRISPR/Cas9 All-in-One plasmid of pSpCas9(BB)-2A-Puro (PX459) v2.0 and pSpCas9(BB)-2A-GFP (PX458) including the LH3 guide RNA (gRNA) sequence for *Plod3* exon 5 (5′-TCCACTGGCGGACAATCTGATGG-3′) and exon 7 (5′-TCGTGTGCGCATCCGGAATGTGG-3′), which were designed and purchased from GenScript, respectively. Two plasmids were transfected into the cells using Lipofectamine 3000 (Life Technologies) according to the manufacturer’s instructions. After 24 h, transfected cells were treated with 10 μg/ml puromycin (Gibco) to select cells containing the PX459 plasmid. After puromycin selection, the GFP-positive cells containing the PX458 plasmid were isolated using a corning ring and cultured them with standard DMEM.

### DNA sequencing

To confirm LH3 gRNA edited *Plod3* exon 5 and 7, PCR products including the region targeted with gRNA were sequenced. DNA was prepared from WT and LH3 KO cells. Cells were plated onto 6 cm dish and grown to 100% confluency. Cells were collected by centrifugation after treating with trypsin (Promega). This cell pellet was digested by 3 mg/ml proteinase K in 20 μl of 50 mM Tris–HCl buffer containing 20 mM NaCl, 1% SDS, and 1 mM EDTA, pH 8.0, at 95 °C for 5 min after preincubating at 60 °C for 60 min. After the digestion, 200 μl of water was added to this solution as a template for PCR. PCR was performed with primer set A (forward: 5′-AAGCGCTTCCTCAACTCTG-3′ and reverse: 5′-TCCCTCATTACCCACCTCTAA-3′) and B (forward: 5′-ACTCCTGAGCATGTGTGTTAG-3′ and reverse 5′-ACAGGGTTGAGTGGCTAAAG-3′) for exon 5 and 7, respectively. Sequencing samples including the forward primer were sent to Quintara Biosciences. Sequencing results were analyzed by GeneStudio Professional Edition, version 2.2.0.0 (GeneStudio, Inc).

### Protein analyses in cell lysate and conditioned culture medium

WT and LH3 KO cells were plated and grown to 80 to 90% confluency. Following stimulation of procollagen biosynthesis with ascorbic acid for 1 day, the medium was replaced to the fresh standard DMEM, and the cells were cultured for 2 days. The medium containing secreted proteins was dissolved in Bolt LDS sample buffer 4× (Life Technologies) with reducing agents. After the cells were washed with PBS once, cell lysates were extracted using M-PER (Thermo Fisher Scientific) containing Halt Protease Inhibitor Cocktail, EDTA-Free (Thermo Fisher Scientific) at 4 °C according to the manufacturer’s instructions. After centrifugation, soluble proteins in the supernatant were mixed with the same sample buffer with reducing agents. These protein solutions were separated on SDS-PAGE gels and then transferred to polyvinylidene difluoride (PVDF) membranes, and Western blots were performed. Gels, buffers, transfer condition, and antibodies are listed in Table S4. Blots were developed with horseradish peroxidase–enhanced Super-Signal West Pico Chemiluminescent Substrate (Thermo Fisher Scientific) and detected by ChemiDoc MP imaging system (Bio-Rad) using the software Image Lab, version 4.0.1 (Bio-Rad). The intensities of protein signals were measured by ImageJ (National Institutes of Health).

### Secretion rate assay for PFHR9 cells

WT and LH3 KO cells were cultured in a 16 cm dish grown to 100% confluency and equally plated to three 6-well tissue culture plates after the cells were trypsinized. Following stimulation of procollagen biosynthesis with ascorbic acid overnight, the cells were preincubated in pulse medium (Met- and Cys-free DMEM with 10% fetal bovine serum and 100 μg/ml ascorbic acid 2-phosphate) without AHA (Click Chemistry Tools) at 37 °C for 30 min to deplete methionine. Pulse labeling was performed with 1 mM AHA in pulse medium at 37 °C for 15 min, and cells were incubated in 1.5 ml chase medium (standard DMEM) at 37 °C for different time intervals (*i.e.*, every 30 min by 360 min, 420 min 480 min, 24 h and 48 h). The conditioned culture medium was collected at each time point and frozen at −20 °C. After thawing and centrifuging at high speed to remove residual cells and large aggregates, 1.4 ml of medium was mixed with 1.4 ml of 0.1 M Tris–HCl containing 1.0 M urea, pH 7.5. This solution was incubated with 0.4 ml of Q Sepharose Fast Flow (GE Healthcare) to remove AHA-incorporated laminins at room temperature for an hour. After centrifugation at high speed for 10 min, 2.8 ml supernatant was mixed with 0.2 ml 10 M acetic acid and 1 ml of 5 M NaCl at 4 °C overnight. Collagen α1α1α2(IV) was precipitated at the highest speed by a tabletop centrifuge. The pellet containing collagen α1α1α2(IV) was dissolved in 27 μl of Tris-buffered saline and labeled with 3 μl of Click-iT Alexa Fluor 488 sDIBO Alkyne (Thermo Scientific) at room temperature for an hour. In LH3 KO secretion rate assay, the pellets from two wells in the 6-well plate were combined to detect adequate signals. This labeled solution was mixed with 10 μl of 4× Bolt LDS sample buffer with reducing agent and separated on precast 6% Tris–glycine gel (Invitrogen). The signals from AHA-Alexa Fluor 488 were detected by the ChemiDoc MP imaging system using the software Image Lab, version 4.0.1. The intensities of fluorescent signals were measured by ImageJ. The AHA-incorporated collagen α1α1α2(IV) as a loading control was purified from conditioned culture medium after an hour pulse and the overnight chase. The purification method is described in the section “Collagen α1α1α2(IV) purification” later. This assay was performed five times, and representative images are shown.

### Secretion rate assay for MEFs

WT and LH3 KO cells were cultured in a 16 cm dish grown to 100% confluency and equally plated to five 10 cm dishes plate after the cells were trypsinized. Following stimulation of procollagen biosynthesis with ascorbic acid overnight, cells were preincubated in pulse medium without AHA at 37 °C for 30 min to deplete methionine. Pulse labeling was performed with 1 mM AHA in pulse medium at 37 °C for 15 min, and cells were incubated in 3.7 ml chase medium (standard DMEM) at 37 °C for different time intervals (*i.e.*, every 30, 60, 90, 120, and 180 min). The conditioned culture medium was collected at each time point and frozen at −20 °C. After thawing and centrifuging at high speed to remove residual cells and large aggregates, 3.5 ml of medium was mixed with 0.5 ml 10 M acetic acid and 20 μl of 100 mg/ml pepsin at 4 °C overnight. After adding 1.0 ml of 5 M NaCl to the pepsin-treated solution and incubating at 4 °C overnight, collagens α1α1α2(I) and α1α1α1(III) were precipitated at the highest speed by a tabletop centrifuge. The pellet containing collagens α1α1α2(I) and α1α1α1(III) was dissolved in 27 μl of Tris-buffered saline and labeled with 3 μl of Click-iT Alexa Fluor 488 sDIBO Alkyne at room temperature for an hour. This labeled solution was mixed with 10 μl of 4× Bolt LDS sample buffer without reducing agent and separated on precast 6% Tris–glycine gel. The signals from AHA-Alexa Fluor 488 were detected by the ChemiDoc MP imaging system using the software Image Lab, version 4.0.1. The intensities of fluorescent signals were measured by ImageJ. The AHA-incorporated type I and type III collagen as a loading control was purified from conditioned culture medium after an hour pulse and the overnight chase. The purification method is described in the section”Collagens α1α1α2(I) and α1α1α1(III) purification” later. This assay was performed five times, and representative images are shown.

### Collagen α1α1α2(IV) purification

Extraction of collagen α1α1α2(IV) was performed using the standard DMEM with PFHR9 cells. DMEM was prepared by the same procedures as described in the aforementioned “Protein level analyses” section, except using serum-free DMEM instead of the standard DMEM. The DMEM was dialyzed with 0.2 M acetic acid at 4 °C overnight. After spinning down to remove residual cells or aggregates, the DMEM was dialyzed with 0.2 M acetic acid containing 1.2 M NaCl at 4 °C overnight. The precipitates including collagen α1α1α2(IV) were collected by centrifugation at 13,000 rpm for 20 min using JA-14 rotor (Beckman) and resuspended with 50 mM Tris–HCl buffer containing 25 mM NaCl and 0.5 M urea, pH 7.5. After dialysis to the same buffer at 4 °C overnight and centrifugation at 13,000 rpm for 10 min using JA-20 rotor (Beckman), the solution was applied to HiTrap Q FF column (cytiva), and the flow through fraction was collected. The flow through was dialyzed to 50 mM Mes buffer containing 25 mM NaCl and 0.5 M urea, pH 6.0, at 4 °C overnight and applied to HiTrap SP HP column (cytiva). After washing with the same Mes buffer at least five column volumes, collagen α1α1α2(IV) was eluted by 50 mM Mes buffer containing 250 mM NaCl and 0.5 M urea, pH 6.0. The fractions containing collagen α1α1α2(IV) were concentrated using Amicon Ultra centrifugal filters Ultracel—100 K (Millipore) at the speed × 100*g* up to 1.5 ml as a total volume. The concentrated fraction was dialyzed to 0.2 M acetic acid at 4 °C overnight, and 0.5 ml of 5 M NaCl was added (final concentration of 1.25 M NaCl) to the dialyzed solution. After mixing at 4 °C overnight, collagen α1α1α2(IV) was precipitated by centrifugation at 14,000 rpm for 20 min using a tabletop centrifuge. The pellet was resuspended with 0.2 M acetic acid dialyzed to 0.2 M acetic acid at 4 °C overnight to remove residual NaCl for experiments.

### Collagens α1α1α2(I) and α1α1α1(III) purification

Extraction of collagens α1α1α2(I) and α1α1α1(III) was performed using the cultured standard DMEM with MEFs. The DMEM was prepared by the same procedures as described in aforementioned “Protein level analyses” section. The DMEM was dialyzed with 0.5 M acetic acid at 4 °C overnight. After spinning down to remove residual cells or aggregates, the DMEM was incubated with pepsin added to a final concentration of 0.25 mg/ml at 4 °C overnight. The pepsin-treated DMEM was dialyzed with 0.5 M acetic acid containing 0.7 M NaCl at 4 °C overnight. The precipitates including collagens α1α1α2(I) and α1α1α1(III) were collected by centrifugation at 13,000 rpm for 20 min using JA-14 rotor. The pellet was resuspended in 0.2 M acetic acid, and, this solution contained enriched collagens. α1α1α2(I) and α1α1α1(III) were dialyzed to 0.2 M acetic acid at 4 °C overnight to remove residual NaCl for experiments.

### SDS-gel analyses

To check purity, the isolated collagens were run on a Bolt 4 to 12% Bis–Tris Plus gel (Invitrogen) in the presence or the absence of DTT with Mes running buffer (Novex) after boiling denaturation. To compare differences in migration, the purified collagens α1α1α2(I) and α1α1α1(III) mixture were run on a 3 to 8% Tris–acetate gel (Invitrogen) in the presence or the absence of DTT with boiling denaturation. To demonstrate higher order collagen α1α1α2(IV) assemblies, SDS-agarose gel electrophoresis was performed for purified WT and LH3 KO collagen α1α1α2(IV). About 3.5% agarose was dissolved in 0.2 M Tris–HCl, pH 8.8. After measuring the total weight of the beaker and agarose solution, the beaker was heated in a microwave oven and gently swirled to thoroughly mix the agarose solution. After adding 20% SDS up to 0.25% and sufficient hot distilled water to obtain the initial weight, the SDS-agarose solution was mixed thoroughly again. Bio-Rad Mini-PROTEAN Tetra Handcast Systems was adapted to cast the gel, and hot SDS-agarose solution was loaded between preheated glass plates of a vertical electrophoresis apparatus. Four different sample conditions were prepared using purified WT and LH3 KO collagen α1α1α2(IV) in the presence or the absence of DTT with and without boiling denaturation. The samples were separated on a SDS—3.5% agarose gels with Tris–glycine SDS running buffer using 75 V constant voltage at 4 °C for about 2 h. All gels were stained with GelCode Blue Stain Reagent (Thermo Scientific). For PTM analysis, we used 6% Tris–glycine SDS-PAGE and 3.0% SDS-agarose gel to separate individual collagen chains of collagens α1α1α2(I) and α1α1α1(III) and collagen α1α1α2(IV) under reducing condition, respectively. After gels were electrophoretically transferred to PVDF membranes with 0.05% SDS, the membranes were stained with Ponceau S.

### Protein identification by LC–MS

Gel bands were reduced with 10 mM DTT at 56 °C for 30 min followed by alkylation with 50 mM iodoacetamide at room temperature for 30 min. The samples were digested with trypsin at 37 °C for 16 h, and the generated tryptic peptides were sequentially extracted from the gels with 5% formic acid, 5% formic acid/50% acetonitrile, and 5% formic acid/95% acetonitrile. The extracted solutions were concentrated by a centrifugal evaporator CVE-3100 (EYELA) and then analyzed by LC–MS on a maXis II quadrupole time-of-flight mass spectrometer (Bruker Daltonics) coupled to a Shimadzu Prominence UFLC-XR system (Shimadzu) with chromatographic separation using an Ascentis Express Peptide ES-C18 column (2.7 μm particle size, L × I.D. 150 mm × 2.1 mm; Supelco) (75). The mass spectrometry (MS) scan and MS/MS acquisition were performed over the *m/z* ranges of 50 to 2500 with a frequency of 5 Hz. The acquired MS/MS spectra were searched against the UniProtKB/Swiss-Prot database (release 2018_05) for *Mus musculus* species using ProteinPilot software 4.5 (AB Sciex), as described previously (24).

### Rotary shadow electron microscopy

Rotary shadowing was performed by methods described previously (76). In brief, samples were prepared to mix WT and LH3 KO collagen α1α1α2(IV) (approximate concentrations of 100 μg/ml) in 0.2 M acetic acid with glycerol to a final concentration of 70% glycerol. Each protein was nebulized onto freshly cleaved mica chips using an airbrush. The samples were then rotary shadowed with carbon/platinum using an electron beam gun within a vacuum evaporator. Images were acquired using an FEI G20 transmission electron microscope. Proportions of different heterotrimer structures were obtained from images of WT (n = 690) and LH3 KO (n = 295) molecules, and representative images were used in Figure 4*C*.

### CD

CD spectra were recorded on an AVIV 202 spectropolarimeter (AVIV Biomedical, Inc) using a Peltier thermostat–controlled cell holder and a 1 mm path length rectangular quartz cell (Starna Cells, Inc). Protein concentrations were determined by acid hydrolysis amino acid analysis described in the section ”PTM analysis by LC–MS” and using collagen solution instead of PVDF membrane. Both WT and LH3 KO collagen α1α1α2(IV) were measured in 0.2 M acetic acid at 10 °C. The denatured WT collagen α1α1α2(IV) was measured using the cuvette after the measurements at 10 °C with heating up to 65 °C for 1 h. The spectra represent the average of at least three scans recorded at a wavelength resolution of 0.1 nm.

### SPR analysis

SPR experiments were carried out using a BIAcore X instrument (GE Healthcare). Purified WT and LH3 KO collagen α1α1α2(IV) were immobilized on a CM5 sensor chip by amide coupling. The approximate coupled protein concentration was 1.2 ng/mm^2^ (1200 response units) and 1.4 ng/mm^2^ (1400 response units) of WT and LH3 KO collagen α1α1α2(IV), respectively. The experiments were performed at 20 °C in 10 mM Hepes, pH 7.4, containing 150 mM NaCl and 0.005% Surfactant P20 using a flow rate of 10 μl/min. All curves are the average of at least three replicates, and three independent measurements were performed. For the analysis of the binding affinity, the curves were fitted with the Langmuir binding model (BIAevaluation software; GE Healthcare). Hsp47 were purified from 17-day-old chicken embryos using methods described previously (77).

### PTM analysis by LC–MS

The hydroxylation rate of Lys (Lys + Hyl = 100%) and Pro (Pro + 4-Hyp + 3-Hyp = 100%) in each α chain of collagens α1α1α2(IV), α1α1α2(I), and α1α1α1(III) was evaluated by LC–MS after acid hydrolysis, as described previously (78). In brief, after respective α chains were transferred to PVDF membranes as described in aforementioned SDS-gel analysis, the membrane bands were excised and subjected to acid hydrolysis (6 N HCl/1% phenol, 110 °C for 20 h in the gas phase under N_2_) after adding stable isotope–labeled collagen (SI-collagen) (79) as an internal standard. The acid hydrolysates were analyzed by LC–MS in multiple reaction monitoring mode on a QTRAP 5500+ hybrid triple quadrupole/linear ion trap mass spectrometer (AB Sciex) coupled to an ExionLC AD HPLC system (AB Sciex) with a ZIC-HILIC column (3.5 μm particle size, L × I.D. 150 mm × 2.1 mm; Merck Millipore). The content of Pro, 3-Hyp, 4-Hyp, Lys, and Hyl was quantitated by the peak area ratio of the analytes relative to the corresponding stable isotopically heavy analytes derived from SI-collagen (79). The occupancy of *O*-glycosylations attached to Hyl (free Hyl + galactosyl Hyl + glucosyl galactosyl Hyl = 100) in the collagens α1α1α2(I) and α1α1α1(III) mixture and purified collagen α1α1α2(IV) was evaluated by LC–MS after alkaline hydrolysis, as described previously (79). In brief, the purified collagen samples were subjected to alkaline hydrolysis (2 N NaOH, 110 °C for 20 h under N_2_) after adding SI-collagen as the internal standard. The content of free Hyl, galactosyl Hyl, and glucosyl galactosyl Hyl was quantitated by LC–MS analysis of the alkaline hydrolysates as described previously.

### Human material and ethics statement

About 113 unrelated fetuses affected by an ICH grade III or IV were included following termination of pregnancy or intrauterine fetal death. They were referred for *COL4A1/COL4A2* screening to the French national molecular genetics reference center for inherited cerebrovascular disorders (Saint Louis Hospital, Paris) and shown to be negative using a targeted high-throughput sequencing (conditions available upon request). Fetal ICH was in most cases detected at systematic second and third trimester ultrasound examinations and in some cases confirmed by fetal MRI and/or pathological examination. Systematic review of medical charts was performed in order to exclude fetuses with an identifiable cause or known risk factor for ICH, including evidence of maternal trauma during pregnancy, cocaine or maternal drug use, maternal or neonatal infections, and fetal alloimmune thrombocytopenia. Written informed consent for genetic investigation and research was provided by parents in accordance with the declaration of Helsinki and the French law. This study has been approved by the Inserm Ethics Committee (INSERM IRB00003888). Genomic DNA was isolated from postmortem fetal tissue and peripheral blood leukocytes of both parents and relatives when available. Prior to whole exome sequencing, we excluded the presence of a mutation in *COL4A1* and *COL4A2* genes by performing a targeted high-throughput sequencing (conditions available upon request).

### Exome sequencing

Exon capture was performed at the IntegraGen platform (Evry) for fetus probands and relatives using the SureSelect Human All Exon V5-UTR (Agilent Technologies) or the Twist Human Core Exome Enrichment System (Twist Bioscience). Followed by 100 base pair paired-end sequencing using an Illumina NovaSeq platform. Data analysis was performed with the IntegraGen in house bioinformatic pipeline. Sequence reads were aligned to the human genome reference GRCh38/hg38 using BWA. Variant calling for the identification of single nucleotide variations, and small insertions/deletions were performed *via* the Broad Institute’s GATK Haplotype Caller GVCF tool (GATK 3.8.1). Ensembl’s VEP (Variant Effect Predictor, release VEP 95.1) program was used to process variants for further annotations. Allele frequency annotations was based on gnomAD (version 2.1.1) and 1000Genomes datasets. PolyPhen-2 algorithm was used to predict deleteriousness of missense variants. Finally, we used CANOES for the detection of copy number variation in whole exome sequencing data (80).

### Qualifying variants in the *PLOD3* gene

Several criteria were used for the selection of qualifying variants in the *PLOD3* gene: (1) quality control: a minimum of 10 reads was required for depth coverage and a minimum of 25% for allelic balance, (2) variants’ nature: all coding and splice site variants were retained, (3) minor allele frequency ≤1/1000 in external databases (gnomAD, version 2.1.1 and 1000Genomes phase 3), and (4) for missense variants, predicted possibly or probably damaging with PolyPhen-2. Investigation for variant enrichment in the *PLOD3* gene was performed by comparing our fetus cohort (N = 113) to cohorts of control individuals in gnomAD v2.1 and v3.1 with the same filter criteria. Burden tests/*p* values were used for statistical analyses.

### Mapping of the amino acid residues in protein structure

The human LH3 crystal structure was retrieved from the Research Collaboratory for Structural Bioinformatics Protein Data Bank (www.rcsb.org, accession number: 6FXK) and used for variant modeling using the UCSF Chimera software (version 1.14; build 42094).

### Statistical analyses

For comparisons between two groups, one-way ANOVA was performed to determine whether differences between groups are significant using OriginPro, version 9.1 (OriginLab Corp). A *p* value of less than 0.05 was considered statistically significant.

## Data availability

All data are contained within the article and supporting information. All source data are available from the corresponding author upon reasonable request. The MS datasets for protein identification of collagen α1α1α2(IV) samples have been deposited to the ProteomeXchange consortium *via* the jPOST partner repository with the dataset identifier PXD035051 (http://proteomecentral.proteomexchange.org/cgi/GetDataset?ID=PXD035051).

(References: https://www.nature.com/articles/nbt.2839 and https://academic.oup.com/nar/article/45/D1/D1107/2605695?login=false).

## Supporting information

This article contains supporting information.

## Conflict of interest

The authors declare that they have no conflicts of interest with the contents of this article.
