# Supplementary material for: Lysyl hydroxylase 3–mediated post-translational modifications are required for proper biosynthesis of collagen α1α1α2(IV)
Source: J Biol Chem. 2022 Nov 17;298(12):102713. doi: 10.1016/j.jbc.2022.102713 (PMC9761383; doi:10.1016/j.jbc.2022.102713)
Supplement: Supporting information [file mmc1.pdf]

Supporting Figures and Tables for

Lysyl hydroxylase 3 mediated post-translational modifications are required for proper biosynthesis of collagen $\alpha1\alpha1\alpha2$(IV)

Yoshihiro Ishikawa[1*], Yuki Taga[2], Thibault Coste[3], Sara F. Tufa[4], Douglas R. Keene[4], Kazunori Mizuno[2], Elisabeth Tournier-Lasserve[3] and Douglas B. Gould[1,5*]

From 1 Department of Ophthalmology, University of California San Francisco, School of Medicine, California, USA, 2 Nippi Research Institute of Biomatrix, Ibaraki, Japan, 3 Université Paris Cité, Inserm, Neurodiderot, F-75019 Paris, France, 4 Research Department, Shriners Hospital for Children, Portland, Oregon, USA, 5 Department Anatomy, Cardiovascular Research Institute, Bakar Aging Research Institute, and Institute for Human Genetics, University of California, San Francisco, California, USA

*For correspondence: Yoshihiro Ishikawa (yoshihiro.ishikawa@uscf.edu) and Douglas Gould (douglas.gould@ucsf.edu)

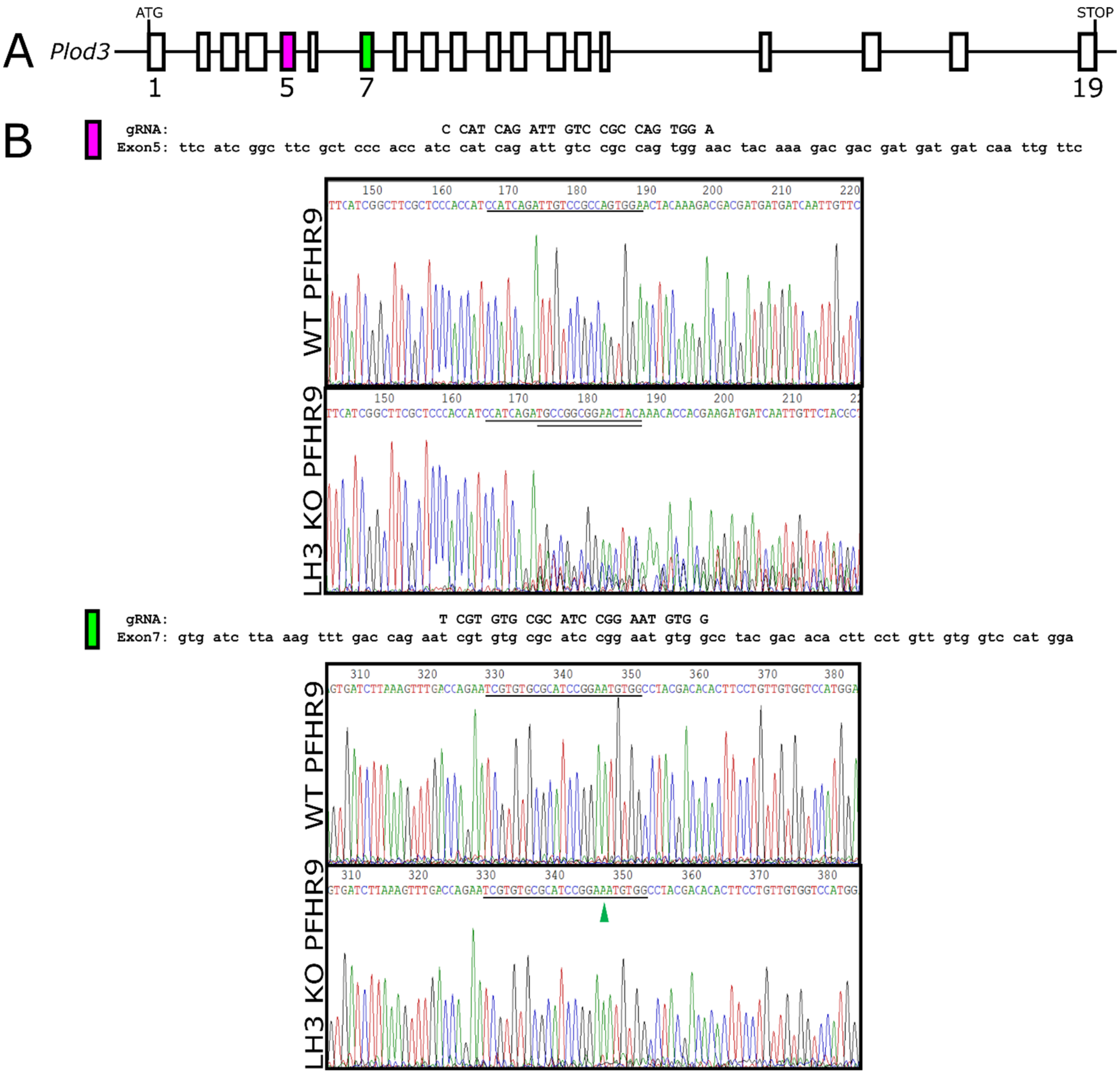

Figure S1. Validation of *Pold3* gene targeting in PFHR9 cells.
(A) *Plod3* exon structure with targeted exons 5 and 7 highlighted in magenta and green, respectively. **(B)** Exon sequence and guide RNA (gRNA) sequences are shown in lower and upper case, respectively. The underlined sequence indicates the gRNA target site and sequencing results showing mixed sequence for exon 5 (starting at double line) indicating that the gRNA edited each strand differently and uniform editing of exon 7 with both strands showing insertion of an adenine (A) nucleotide (green arrowhead).

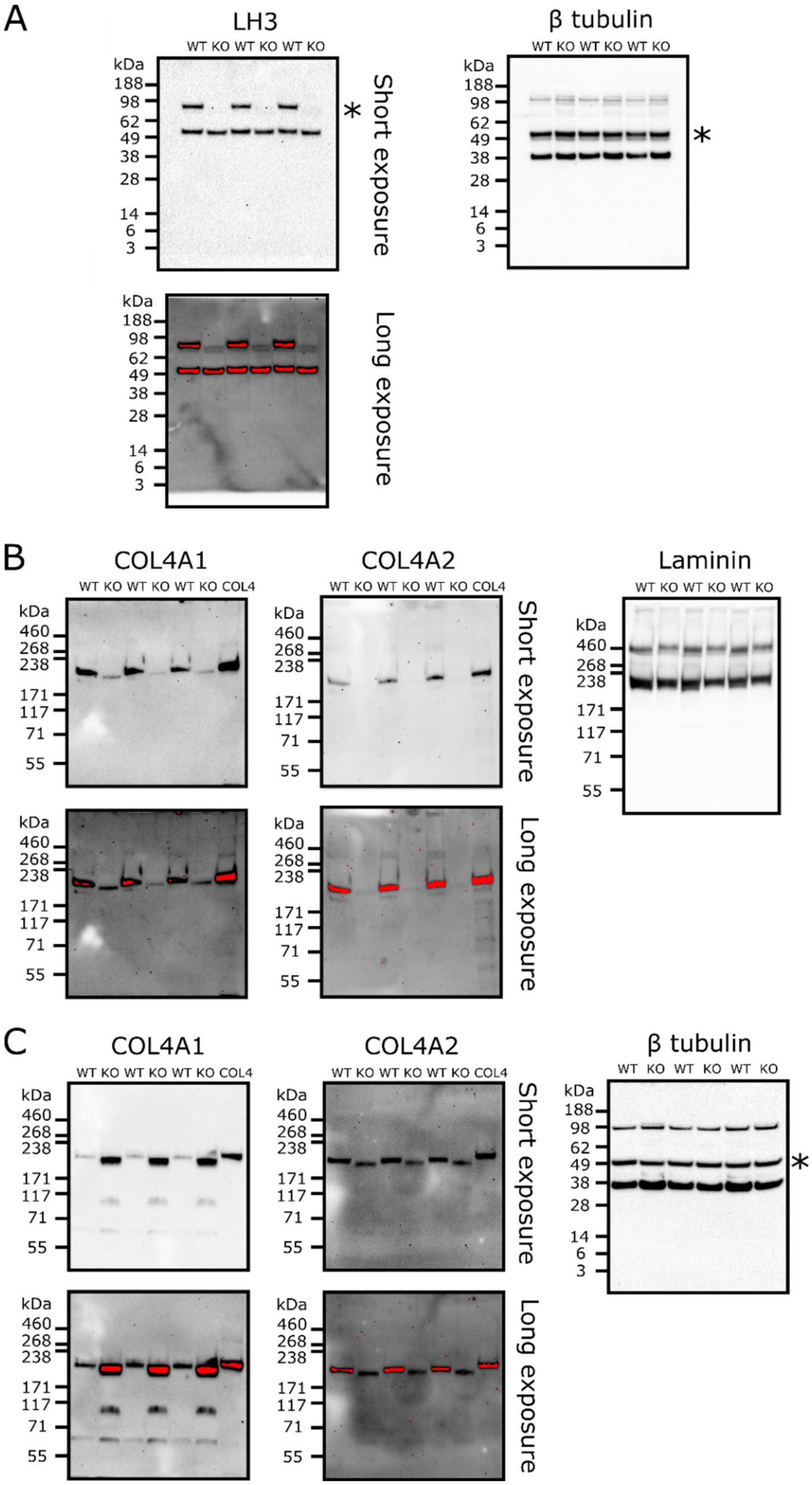

Figure S2. Relative protein levels of LH3 and collagen α1α1α2(IV) in WT and LH3 KO PFHR9 cells. Uncropped images of Western blots presented in Figure 1 to confirm **(A)** LH3 KO and compare **(B)** extracellular and **(C)** intracellular COL4A1 and COL4A2 levels in WT and LH3 KO PFHR9 cells**.** The asterisk indicates the migration corresponding to the molecular weight of the protein, which was used for further quantitative analysis. The red color on the gel bands in Long exposure images indicates saturated signals and overexposure. COL4 indicates control purified collagen α1α1α2(IV).

Table S1. Rate of secretion for collagen α1α1α2(IV) in WT and LH3 KO PFHR9 cells

	collagen α1α1α2(IV) (n = 5)	
time (min)	WT	KO
30	0.07 ± 0.02	NM
60	0.15 ± 0.04	NM
90	0.23 ± 0.07	NM
120	0.28 ± 0.07	NM
150	0.34 ± 0.11	NM
180	0.37 ± 0.08	NM
210	0.46 ± 0.10	NM
240	0.60 ± 0.06	NM
270	0.73 ± 0.07	NM
300	0.77 ± 0.14	NM
360	0.94 ± 0.12	NM
420	0.99 ± 0.11	NM
480 / 8 h	1	0.03 ± 0.01
1440 / 24 h	NM	0.13 ± 0.03
2880 / 48 h	NM	0.24 ± 0.13

NM: Not Measured

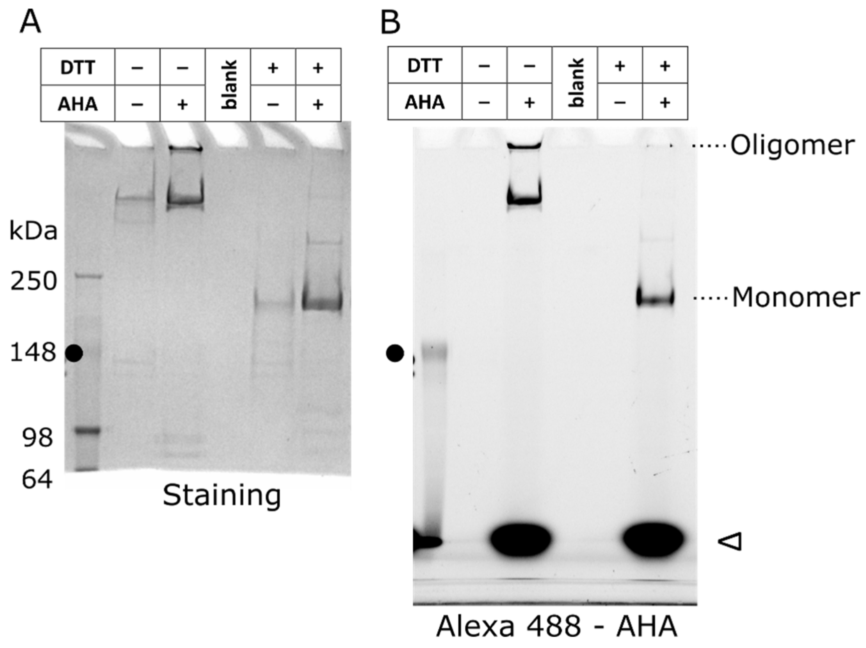

Figure S3. SDS-PAGE analysis of control AHA-incorporated collagen α1α1α2(IV) purified from the conditioned media of PFHR9 cells.

SDS-PAGE analysis of purified control AHA-incorporated collagen α1α1α2(IV) using a 6 % Tris-glycine gel in the presence and absence of a reducing agent (DTT). The gel images are **(A)** stained with GelCode Blue Stain Reagent and **(B)** detect AHA-Alexa Fluor 488. Arrowhead indicates dye front showing un-incorporated AHA.

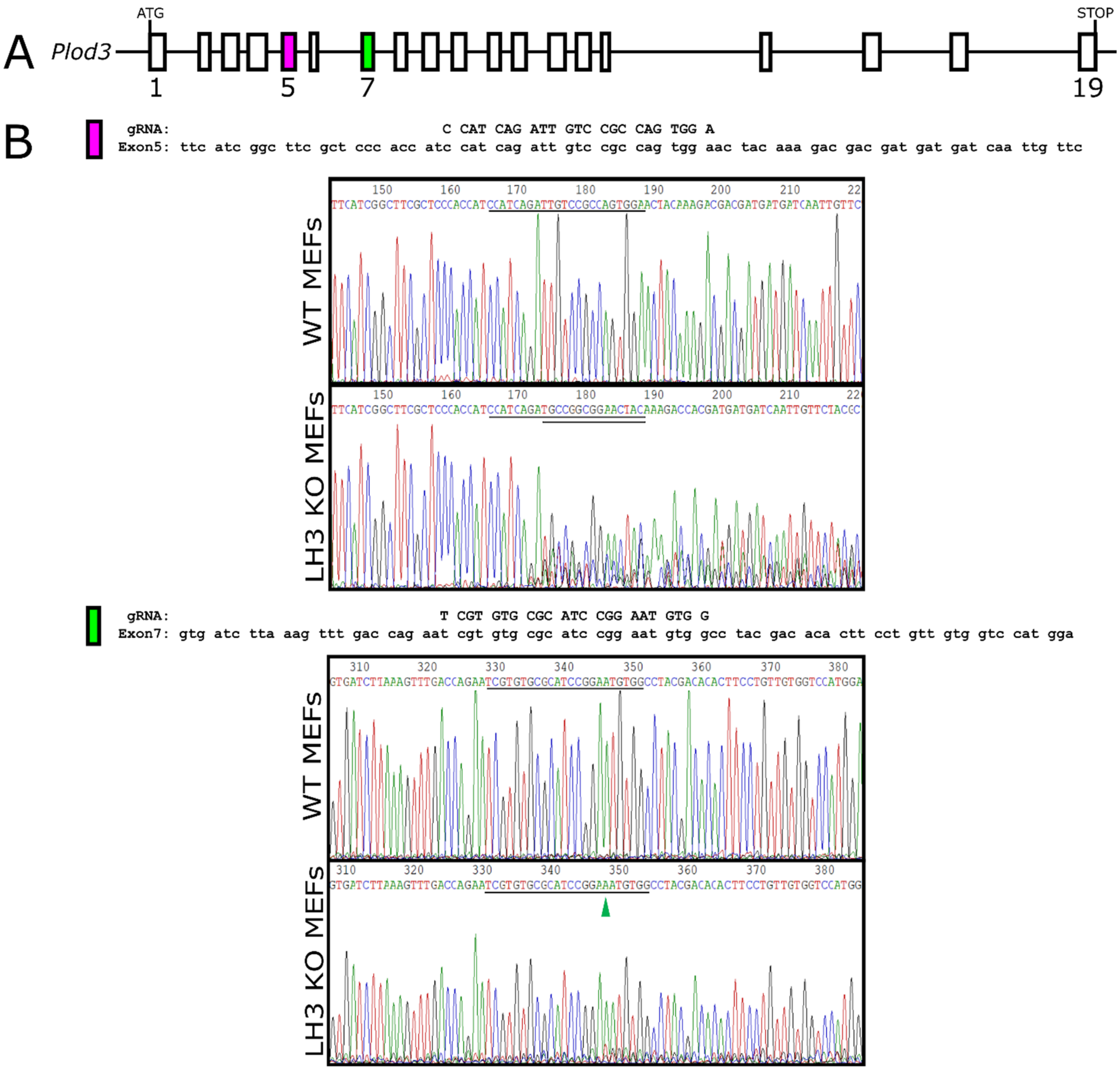

Figure S4. Validation of *Pold3* gene targeting in MEFs.
(A) *Plod3* exon structure with targeted exons 5 and 7 highlighted in magenta and green, respectively. **(B)** Exon sequence and guide RNA (gRNA) sequences are shown in lower and upper case, respectively. The underlined sequence indicates the gRNA target site and sequencing results showing mixed sequence for exon 5 (starting at double line) indicating that the gRNA edited each strand differently and uniform editing of exon 7 with both strands showing insertion of an adenine (A) nucleotide (green arrowhead).

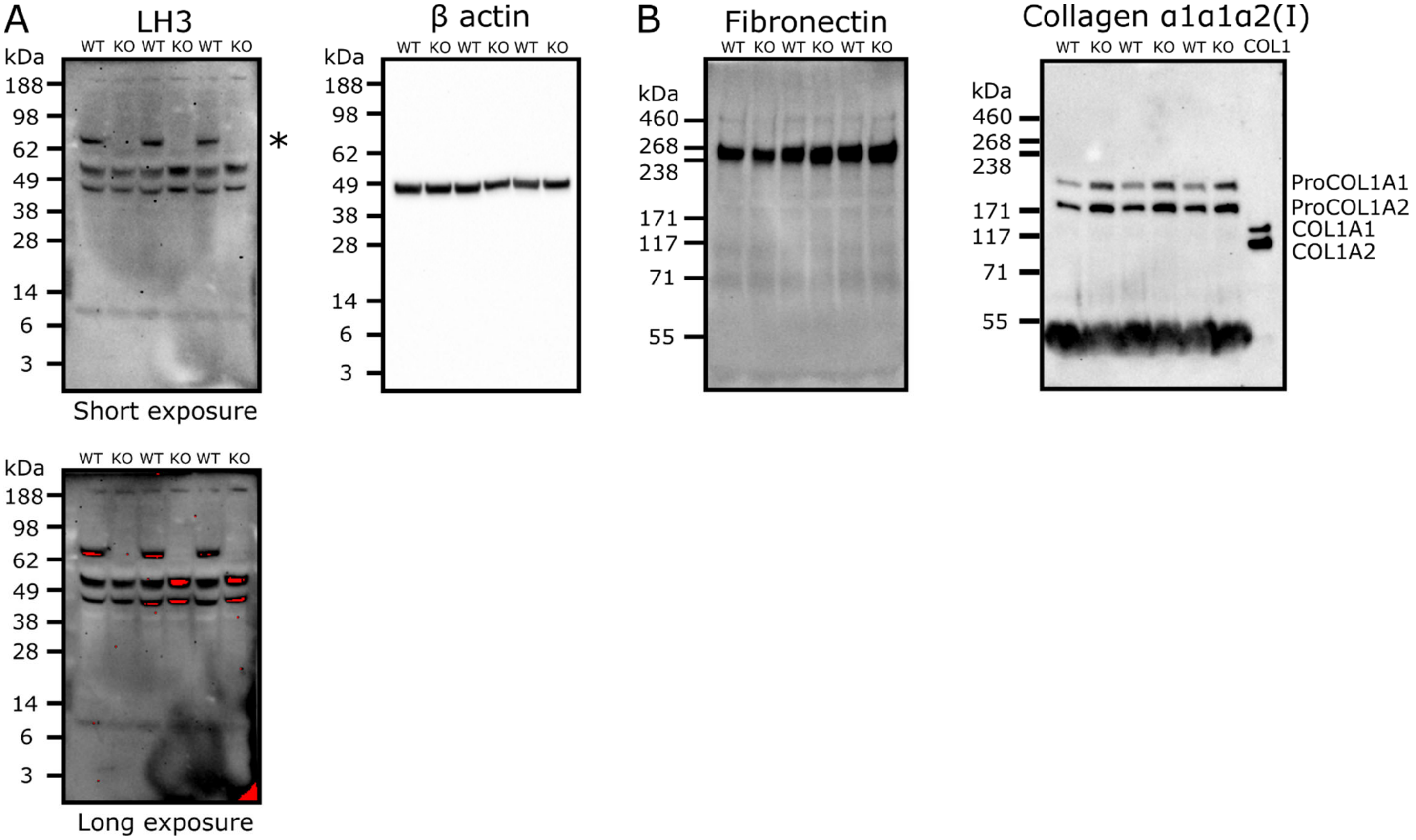

Figure S5. Relative protein levels of LH3 and collagen α1α1α2(I) in WT and LH3 KO MEFs.
Uncropped images of Western blots presented in Figure 2 to confirm **(A)** LH3 KO and compare **(B)** extracellular COL1A1 and COL1A2 levels in WT and LH3 KO MEFs. The asterisk indicates the migration corresponding to the molecular weight of the protein, which was used for further quantitative analysis. The red color on the gel bands in Long exposure image indicates saturated signals and overexposure. COL1 indicates control purified pepsin treated collagen α1α1α2(I).

Table S2. Rate of secretion for collagen α1α1α2(I) and α1α1α1(III)in WT and LH3 KO MEFs

	collagen α1α1α2(I) (n = 5)			collagen α1α1α1(III) (n = 5)		
time (min)	WT	KO	*P* value	WT	KO	*P* value
30	0.44 ± 0.12	0.29 ± 0.02	0.047	0.42 ± 0.09	0.45 ± 0.18	0.79
60	0.75 ± 0.05	0.48 ± 0.08	0.001	0.63 ± 0.21	0.63 ± 0.14	0.97
90	0.87 ± 0.09	0.55 ± 0.08	0.002	0.69 ± 0.19	0.65 ± 0.11	0.69
120	0.94 ± 0.15	0.83 ± 0.15	0.339	0.81 ± 0.15	0.82 ± 0.09	0.85
180	1	0.92 ± 0.15	0.303	1	093 ± 0.12	0.29

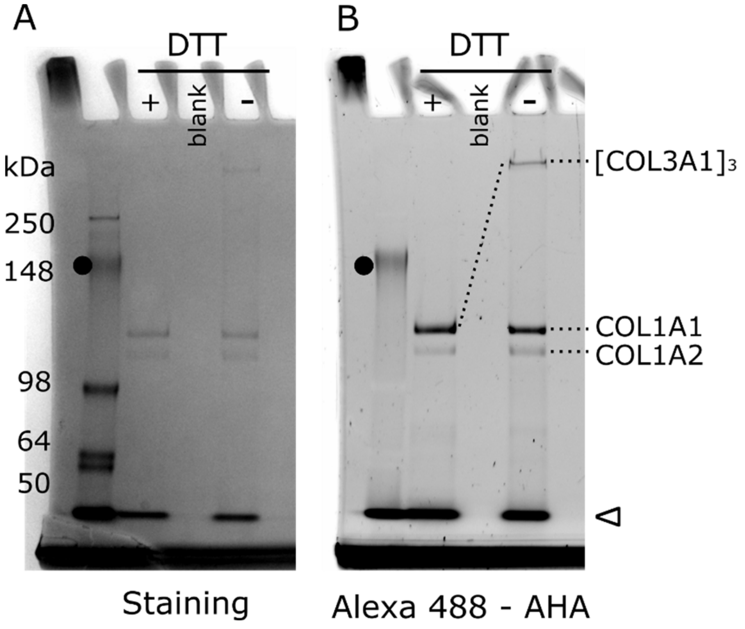

Figure S6. SDS-PAGE analysis of control AHA-incorporated collagens α1α1α2(I) and α1α1α1(III) purified from the conditioned media of MEFs.

SDS-PAGE analysis of control AHA-incorporated a mixture of pepsin treated collagens α1α1α2(I) and α1α1α1(III) using a 6 % Tris-glycine gel in the presence or absence of a reducing agent (DTT). The gel images are **(A)** stained with GelCode Blue Stain Reagent and **(B)** detect AHA-Alexa Fluor 488. Arrowhead indicates dye front including un-incorporated AHA.

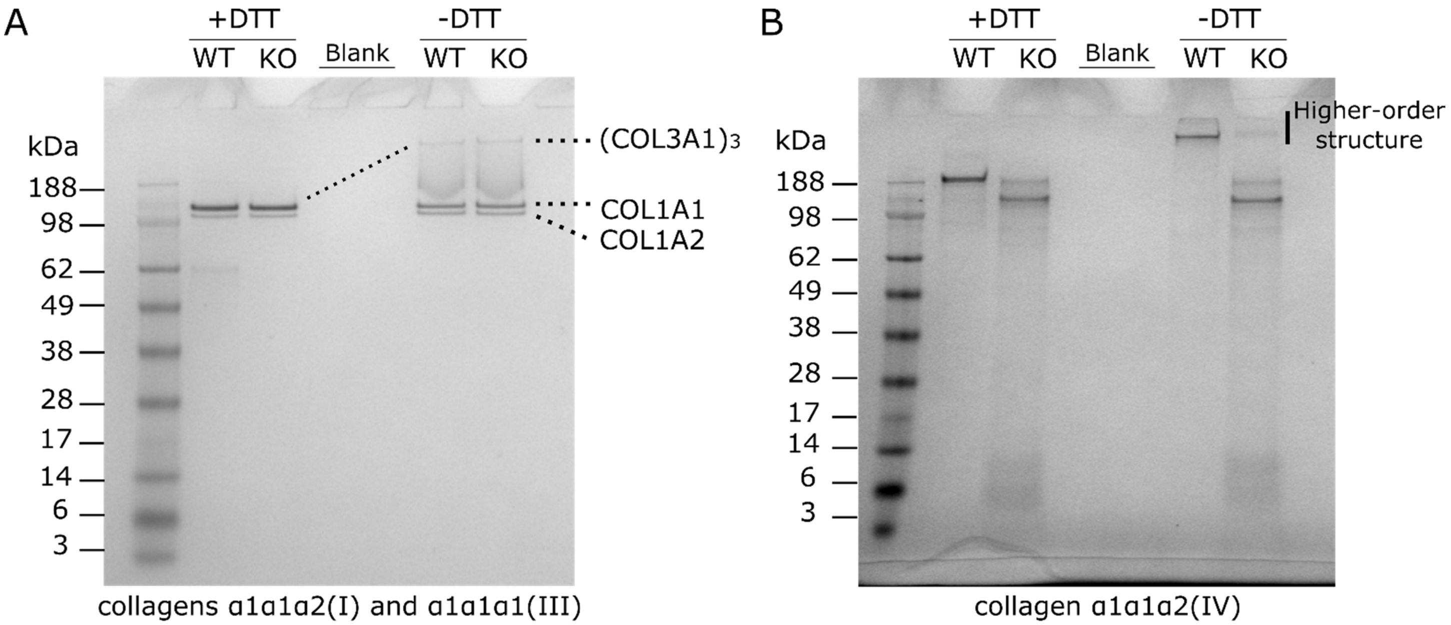

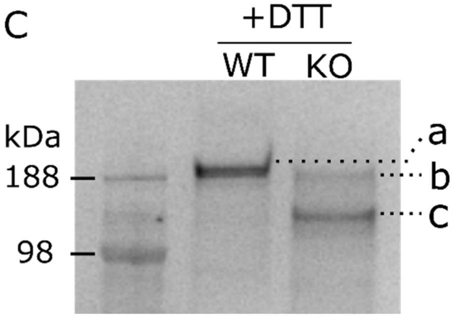

D

band	Score	Coverage (%)	Name	Species
a	16.95	22.8	Collagen alpha-2(IV) chain	MOUSE
	13.66	21.3	Collagen alpha-1(IV) chain	MOUSE
b	11.41	14.1	Collagen alpha-2(IV) chain	MOUSE
	4.41	9	Collagen alpha-1(IV) chain	MOUSE
c	11.39	17.9	Collagen alpha-2(IV) chain	MOUSE
	7.54	13.8	Collagen alpha-1(IV) chain	MOUSE

Figure S7. Overall purity and protein identification of collagens.
Overall purity of **(A)** a mixture of pepsin treated collagens α1α1α2(I) and α1α1α1(III) and **(B)** purified collagen α1α1α2(IV) from conditioned culture media. The purified collagens were run on a Bolt 4 -12 % Bis-Tris Plus gel in the presence or absence of DTT and stained with GelCode Blue Stain Reagent. **(C)** Close up of a lower molecular weight band beneath the collagen α1α1α2(IV) monomer in LH3 KO cells. **(D)** Table showing results from LC-MS analysis to identify the proteins in gel bands a, b, and c from (C). Gel bands were cut from the α1α1α2(IV) gel under the reducing conditions. Keratin was excluded from the list.

Table S3: Comparison of proline and lysine post-translational modifications between of collagens produced by WT and LH3 KO cells.

		3Hyp (%)	4Hyp (%)	Pro (%)	Hyl (%)	Lys (%)
COL4A1	WT (3)	1.7 ± 0.1	**62.7 ± 0.6**	**35.6 ± 0.6**	**81.3 ± 1.1**	**18.7 ± 1.1**
	KO (3)	1.5 ± 0.6	**44.6 ± 2.2**	**53.9 ± 1.6**	**40.3 ± 4.0**	**59.7 ± 4.0**
COL4A2	WT (3)	**1.7 ± 0.1**	*59.0 ± 0.4*	*39.3 ± 0.3*	**76.9 ± 1.5**	**23.1 ± 1.5**
	KO (3)	**2.4 ± 0.1**	*54.5 ± 1.4*	*43.1 ± 1.4*	**56.7 ± 4.0**	**43.3 ± 4.0**
COL1A1	WT (4)	*1.1 ± 0.2*	50.3 ± 0.7	48.6 ± 0.9	30.1 ± 2.3	69.9 ± 2.3
	KO (4)	*0.6 ± 0.1*	50.9 ± 0.2	48.5 ± 0.2	31.7 ± 1.0	68.3 ± 1.0
COL1A2	WT (4)	**0.7 ± 0.2**	*48.8 ± 0.7*	*50.5 ± 0.8*	*36.9 ± 4.0*	*63.1 ± 4.0*
	KO (4)	**0.2 ± 0.1**	*46.5 ± 1.0*	*53.3 ± 1.0*	*29.9 ± 3.3*	*70.1 ± 3.3*
COL3A1	WT (4)	*0.4 ± 0.2*	**58.6 ± 0.2**	**41.0 ± 0.3**	**35.9 ± 1.3**	**64.1 ± 1.3**
	KO (4)	*0.1 ± 0.1*	**57.5 ± 0.4**	**42.4 ± 0.5**	**30.4 ± 0.9**	**69.6 ± 0.9**

COL4A1/COL4A2 and COL1A1/COL1A2/COL3A1 were purified from the culture medium of the PFHR9 and MEFs, respectively.
Values are given as means ± S.D. *Italic* and **Bold** fonts indicate P < 0.05 and P < 0.005, respectively.
The number in the bracket next to the genotypes indicates biological replicates.
3Hyp + 4Hyp + Pro = 100 % and Hyl + Lys = 100 %.
Values of amino acids were obtained using acid hydrolysis amino acid analysis.
3Hyp; 3-hydroxyproline, 4Hyp; 4-hydroxyproline, Pro; proline, Hyl; hydroxylysine, Lys; lysine.

Collagen α1α1α2(IV)

<table>
<tr><td rowspan="2">WT</td><td colspan="6">Total Hyl (%): 79.1 ± 1.3</td><td>Lys (%): 20.9 ± 1.3</td></tr>
<tr><td>93.7 ± 1.3</td><td rowspan="3">P-value: 3.00·E-6</td><td>3.2 ± 0.7</td><td rowspan="3">P-value: 1.77·E-5</td><td>3.1 ± 0.7</td><td rowspan="3">P-value: 0.12</td><td rowspan="3"></td></tr>
<tr><td rowspan="3">KO</td><td>GGHL (%)</td><td>GHL (%)</td><td>Hyl (%)</td></tr>
<tr><td>8.5 ± 3.7</td><td>85.3 ± 5.9</td><td>6.2 ± 2.6</td></tr>
<tr><td colspan="6">Total Hyl (%): 48.5 ± 3.9</td><td>Lys (%): 51.5 ± 3.9</td></tr>
</table>

Mixture of collagens α1α1α2(I) and α1α1α1(III)

<table>
<tr><td rowspan="2">WT</td><td colspan="6">Total Hyl (%): 34.3 ± 1.7</td><td>Lys (%): 65.7 ± 1.7</td></tr>
<tr><td>18.6 ± 1.8</td><td rowspan="3">P-value: 7.80·E-5</td><td>4.8 ± 0.3</td><td rowspan="3">P-value: 5.51·E-7</td><td>76.5 ± 1.5</td><td rowspan="3">P-value: 0.23</td><td rowspan="3"></td></tr>
<tr><td rowspan="3">KO</td><td>GGHL (%)</td><td>GHL (%)</td><td>Hyl (%)</td></tr>
<tr><td>1.8 ± 0.2</td><td>23.0 ± 0.5</td><td>75.2 ± 0.5</td></tr>
<tr><td colspan="6">Total Hyl (%): 30.7 ± 1.4</td><td>Lys (%): 69.3 ± 1.4</td></tr>
</table>

Figure S8. Occupancy (%) of *O*-glycosylation in total hydroxylysine residues in collagens produced by WT and LH3 KO cells. Quantification of the occupancy of *O*-glycosylation attached to hydroxylysine (GGHL + GHL + Hyl = 100 %) in collagen α1α1α2(IV) (top) and mixture of collagens α1α1α2(I) and α1α1α1(III) (bottom) produced by WT and LH3 KO cells shown in Figure 4B. The top and bottom rows in the tables show the ratio of total PTMs in lysine (Hyl + Lys = 100 %), and the values of total hydroxylysine and lysine in collagen heterotrimers were calculated using Table S3. The number of biological replicates is n = 3 for both genotypes and collagens. Data are presented as mean ± S.D. P values are shown in the table. [GGHL; glucosyl galactosyl hydroxylysine, GHL; galactosyl hydroxylysine, Hyl; hydroxylysine, Total Hyl; hydroxylysine with and without *O*-glycosylation, Lys; lysine]. The same colors as Figure 4 are used.

Table S4: List of antibodies, gels, gel running buffers and transfer conditions used for Western blot analyses.

Primary antibodies						
**Name**	**company**	**Product number**	**dilution ratio**	**Gel**	**Gel running buffer**	**with 0.05 % SDS during transferring to PVDF**
anti-LH3 Rabbit Polyclonal	proteintech	11027-1-AP	1:1000	4-12 % Bis-Tris	MES	
anti-β Tubulin III Rabbit Polyclonal	Sigma-Aldrich	T2200	1:1000	4-12 % Bis-Tris	MES	
anti- β Actin Mouse Monoclonal	Santa Cruz Biotech	sc-69879	1:1000	4-12 % Bis-Tris	MES	
anti-Col4a1 NC1 Rat Monoclonal (Clone H11)	Chondrex	7070	1:1000	6 % Tris-Glycine	Tris-Glycine	YES
anti-Col4a2 NC1 Rat Monoclonal (Clone H22)	Chondrex	7071	1:500	6 % Tris-Glycine	Tris-Glycine	YES
anti-Laminin 1+2 antibody Rabbit Polyclonal	abcom	ab7463	1:1000	6 % Tris-Glycine	Tris-Glycine	YES
anti-Mouse Collagen Type I Rabbit Polyclonal	Millipore	AB765P	1:1000	6 % Tris-Glycine	Tris-Glycine	YES
anti-Human Fibronectin Sheep Polyclonal	R&D systems	AF1918	1:1000	6 % Tris-Glycine	Tris-Glycine	YES

Secondary antibodies			
**Name**	**company**	**Product number**	**dilution ratio**
HRP-conjugated anti-rabbit IgG	GE Healthcare	NA934	1:30,000
HRP-conjugated anti-Rat IgG	Thermo Scientific	31470	1:30,000
HRP-conjugated anti-sheep IgG	abcom	ab6747	1:30,000
HRP-conjugated anti-mouse IgG	Dako	P0447	1:30,000
